# Laser-Induced Pd-PdO/rGO Catalysts for Enhanced Electrocatalytic Conversion of Nitrate into Ammonia

**DOI:** 10.1021/acsami.4c06378

**Published:** 2024-07-04

**Authors:** James Ebenezer, Aneena Lal, Parthiban Velayudham, Arie Borenstein, Alex Schechter

**Affiliations:** † Department of Chemical Sciences, Ariel University, Ariel 40 700, Israel; ‡ Research and Development Centre for Renewable Energy, New Technology Centre, University of West Bohemia, 301 00 Pilsen, Czech Republic

**Keywords:** laser processing, nitrate reduction, nitrite, hydroxylamine, bifunctionality, environmental remediation

## Abstract

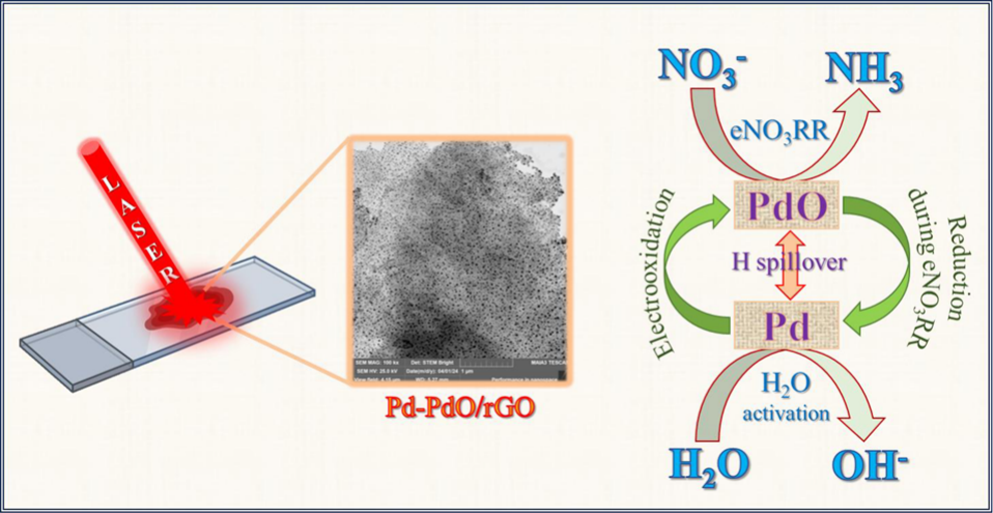

Electrochemical reduction of nitrate to ammonia (eNO_3_RR) is proposed as a sustainable solution for high-rate ammonia synthesis under ambient conditions. The complex, multistep eNO_3_RR mechanism necessitates the use of a catalyst for the complete conversion of nitrate to ammonia. Our research focuses on developing a novel Pd-PdO doped in a reduced graphene oxide (rGO) composite catalyst synthesized via a laser-assisted one-step technique. This catalyst demonstrates dual functionality: palladium (Pd) boosts hydrogen adsorption, while its oxide (PdO) demonstrates considerable nitrogen adsorption affinity and exhibits a maximum ammonia yield of 5456.4 ± 453.4 μg/h/cm^2^ at −0.6 V vs reversible hydrogen electrode (RHE), with significant yields for nitrite and hydroxylamine under ambient conditions in a nitrate-containing alkaline electrolyte. At a lower potential of −0.1 V, the catalyst exhibited a minimal hydrogen evolution reaction of 3.1 ± 2.2% while achieving high ammonia selectivity (74.9 ± 4.4%), with the balance for nitrite and hydroxylamine. Additionally, the catalyst’s stability and activity can be regenerated through the electrooxidation of Pd.

## 1 Introduction

The escalating energy demands and rising environmental pollution from the extensive use of fossil fuels have stimulated research into alternative, cleaner, and more efficient energy conversion and storage technologies.^1,2^ Ammonia (NH_3_) is emerging as one of the viable carbon-free energy carriers due to its high energy density (5.52 kWh/kg) and significant hydrogen content (17.65 wt %).^3^ Currently, the industrial production of ammonia is predominantly carried out via the energy-intensive Haber–Bosch process (HBP), which synthesizes ammonia from hydrogen and nitrogen at high temperatures and pressures using Fe-based catalysts.^4^ The low energy efficiency, substantial carbon dioxide emissions, and reliance on hydrogen feedstock in the HBP have prompted researchers to investigate alternative, more energy-efficient methods for ammonia synthesis.^4^ Meanwhile, nitrate contamination in water seriously threatens human health and aquatic ecosystems.^5^ Addressing both issues, the electrochemical conversion of nitrate to ammonia offers a dual benefit: it provides a potential pollution remedy and a greener approach to ammonia production.

Electrochemical nitrate reduction to ammonia (eNO_3_RR) is a complex process involving an eight-electron transfer that proceeds through multiple reduction steps. This reaction can yield a variety of intermediates and products,^6,7^ as explained in eqs 2–6.
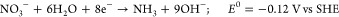
1

The conversion of nitrate to ammonia can occur via direct and indirect pathways, depending on the nitrate concentration and the solution’s pH.^8^ High nitrate concentrations generally favor the indirect reduction pathway,^8^ as demonstrated from eqs 2 to 6. In this scenario, the initial step involves the adsorption of nitrate onto the electrocatalyst surface, which serves as the rate-determining step.^9^ Subsequently, nitrate is reduced to nitrite, followed by further reductions through intermediates such as NO and NH_2_OH, ultimately yielding ammonia.
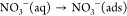
2
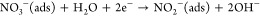
3
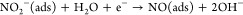
4
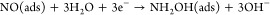
5
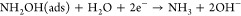
6

The multistep reduction process of nitrate to ammonia, involving stable intermediates such as NO_2_^–^, NH_2_OH, and NH_3_, underscores the critical need for catalysts that can effectively facilitate the complete conversion of nitrate to ammonia. To meet this challenge, a diverse array of catalysts has been developed for electrochemical nitrate reduction reactions, each tailored to enhance the efficiency and selectivity of the process. These catalysts span a wide range of materials, including metal oxides,^10,11^ phosphides,^12,13^ sulfides,^14^ carbides,^15^ alloys,^16,17^ and metal–organic frameworks.^18,19^

Palladium (Pd) stands out among various noble metals for its excellent hydrogen adsorption properties.^20^ A study by Han et al.^21^ highlighted the superior eNO_3_RR performance of Pd nanocrystals, particularly noting that the Pd(111) facet demonstrated exceptional activity with a faradaic efficiency (FE) of 79.9% and an ammonia production rate of 0.55 mmol/h/cm^2^. These figures surpassed the values of Pd(100) and Pd(110) by 1.4 and 1.9 times, respectively. Insights from density functional theory (DFT) analysis attributed the enhanced performance of Pd(111) to the minimal free energy change in the critical step of the reaction (*NH_3_ to NH_3_) and effective suppression of the hydrogen evolution reaction (HER). This research offers insightful perspectives on leveraging Pd-based nanocatalysts and potentially other nanomaterials with optimally oriented facets for eNO_3_RR. In another case, Guo et al.^22^ introduced and developed a zinc-nitrate battery system employing Pd-doped TiO_2_ nanoarrays on carbon cloth as the catalytic electrode, achieving a power density of 0.87 mW/cm^2^ and a remarkable NH_3_ FE of 81.3%. The inclusion of Pd in the TiO_2_ nanoarrays, when used directly as a NO_3_RR catalyst, led to diminished adsorption of intermediates, yet the catalyst delivered an impressive NH_3_ yield of 1.1 mg/cm^2^/h, with an NH_3_ FE of 92.1% and a NO_3_^–^ conversion of 99.6%.

Wang et al.^23^ designed a dual-site eNO_3_RR catalyst by integrating oxygen vacancy-rich MnO_2_ nanosheets with Pd nanoparticles onto a three-dimensional porous nickel foam, creating a Pd–MnO_2_–O_v_/Ni foam catalyst. Employed in a flow cell, this catalyst achieved a NO_3_^–^ conversion rate of 6.42 mg/cm^2^/h from a 22.5 mg/L NO_3_^–^ solution at a flow rate of 0.875 mL/min, with an NH_3_ selectivity of 87.6%. This performance notably surpassed that of MnO_2_–O_v_/Ni and Pd/Ni, underscoring the synergetic effect of oxygen vacancies in MnO_2_–O_v_’s ability to adsorb, stabilize, and activate NO_3_^–^ and nitrogen intermediates like *NOH, *N, and *NH, while Pd sites facilitated hydrogen adsorption essential for eNO_3_RR and oxygen vacancy regeneration. As detailed in this report, the nitrate reduction mechanism is divided into two primary stages: deoxygenation and hydrogenation,^24^ necessitating two distinct active sites for hydrogen adsorption and another for nitrate adsorption. Palladium is recognized for its excellent hydrogen adsorption properties,^25^ which can be complementarily paired with its oxide form, PdO, which is known for its superior nitrate adsorption capabilities compared to Pd. This combination yields a bifunctional catalyst system optimized for eNO_3_RR.

This palladium/palladium oxide (Pd/PdO) catalyst performance can be enhanced with a proper support material that facilitates uniform distribution of active sites. Reduced graphene oxide (rGO), characterized by its two-dimensional sp^2^-bonded carbon atom networks, offers a high surface area and remarkable chemical and electrochemical stability across diverse electrolytic environments.^26^ These properties make rGO an excellent substrate for the uniform deposition of metal/metal oxide nanoparticles, leading to improved electrochemical performance.^27^ Consequently, employing rGO to support well-dispersed Pd/PdO nanoparticles is a promising strategy for enhancing the electrocatalytic reduction of the NO_3_^–^ neighborhood.

Recently, laser processing has emerged as an innovative, cost-effective, and environmentally friendly technique to fabricate rGO films.^28,29^ This method enables the precise scribing of graphitic films onto various substrates in any desired shape. One-step laser irradiation of a mixture containing carbon and metal ions can yield a composite with highly dispersed nanoparticles. Furthermore, the fast thermal reaction of the laser allows the formation of multiphase products, including metallic and various oxide crystals of the processed metal.^30^ Drawing on these developments, we adopted a laser-assisted one-step approach to synthesize a heterostructure of Pd and PdO supported on rGO. Subsequent heat treatment of this composite enhanced the PdO content, leading to a catalyst system that demonstrates superior performance. This heat-treated Pd-PdO/rGO composite catalyst achieved a maximum ammonia yield rate of 5456.4 ± 453.4 μg/h/cm^2^ at −0.6 V vs reversible hydrogen electrode (RHE), alongside yields of 7381.1 ± 594.7 μg/h/cm^2^ for nitrite and 1175.3 ± 204.4 μg/h/cm^2^ for hydroxylamine. At −0.1 V, the system exhibited a minimal hydrogen evolution reaction at 3.1 ± 2.2%, with an ammonia selectivity of 74.9 ± 4.4%, and the remainder was attributed to nitrite (6.4 ± 0.4%) and hydroxylamine (15.6 ± 1.1%). Moreover, the catalyst’s stability could be restored through the electrooxidation of Pd, which was reduced during the reaction process. This approach significantly enhances the efficiency of the electrochemical reduction of nitrate to ammonia, demonstrating its potential for more effective solutions.

## 2 Materials and Methods

Sodium salicylate (C_7_H_5_NaO_3_), potassium hydroxide (KOH), potassium nitrate (KNO_3_), graphite, sodium nitroprusside dihydrate, and ammonium chloride (NH_4_Cl), along with various acids, were sourced from Sigma-Aldrich. Trisodium citrate (Na_3_C_6_H_5_O_7_), sodium hydroxide (NaOH), palladium nitrate hydrate (Pd(NO_3_)_2_·6H_2_O), potassium permanganate (KMnO_4_), and polyacrylonitrile (PAN) were acquired from Merck. Isopropyl alcohol was obtained from Bio-Lab Ltd. in Jerusalem, and sodium hypochlorite solution, with an 11–15 wt % available chlorine content, was purchased from Thermo Scientific. The Nafion 115 membrane and Nafion ionomer, which is a 5 wt % solution in a mixture of lower aliphatic alcohols and water, were procured from the Fuel Cell Store. All chemicals were utilized in their received condition, and ultrapure water with a resistivity of 18.2 MΩ cm was employed for all experimental procedures.

### 2.1 Synthesis of Graphene Oxide (GO)

Graphene oxide (GO) is synthesized by improved Hummer’s method. One gram of graphite pellets of size less than 150–160 μm was added to the H_2_SO_4_ and H_3_PO_4_ acid mixture in a ratio of 9:1 under continuous stirring at 35–40 °C. After the complete dispersion of graphite pellets, 6 g of potassium permanganate (KMnO_4_) was slowly added, considering the exothermic nature of the reaction. The temperature of the reaction mixture is raised to 50–70 °C, and stirring is continued overnight. Quenching of the reaction is proceeded by cooling the reaction mixture to room temperature, followed by the addition of 100 mL of deionized water dropwise in the ice-cold medium. Thirty milliliters of hydrogen peroxide (H_2_O_2_, 30%) is added after the elimination of the excess amount of KMnO_4_.^31^ The brownish-yellow precipitate formed is collected and centrifuged to remove the unreacted graphite. Soxhlet washing and dialysis are conducted for further purification.

### 2.2 Synthesis of the Pd-PdO/rGO Composite

**Scheme 1:**
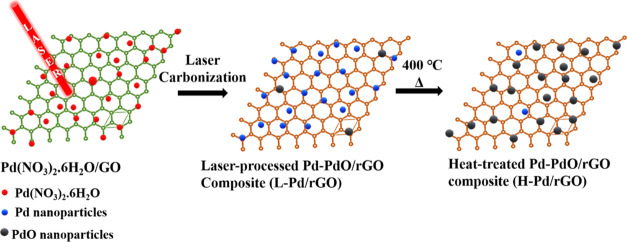
Schematic Illustration of the L-Pd/rGO and H-Pd/rGO Composite Synthesis

Scheme 1 demonstrates the schematic illustration for the synthesis of the Pd-PdO/rGO composite. The absorbed laser energy causes GO and palladium nitrate hydrate (Pd(NO_3_)_2_·6H_2_O) to underlie reduction and produce reduced graphene oxide (rGO) and Pd, respectively. The elevated temperature produced by laser irradiation facilitates the thermal decomposition of metal salt, thus forming a minute quantity of PdO in the composite. The slurry is prepared by mixing dry GO with 100 μL of polyacrylonitrile overnight to form a uniform mixture, followed by the addition of 50 mg of Pd(NO_3_)_2_·6H_2_O. The slurry is kept stirring for 24 h. The uniformly dispersed slurry is cast over the electrode and dried until the excess solvent evaporates. Laser processing of the dried surface turns the Pd-PdO/rGO electrocatalyst. 1.75 W CO_2_ laser (25 W Gravograph Laser engraver, LS100, France) at a scan rate/speed of 20 mm/s is the parameter for the laser processing of the Pd-PdO/rGO catalyst. After laser treatment, the sample was heated to 400 °C under atmospheric air for 2 h with a 5 °C/min heating ramp. The heat-treated sample is used as an electrocatalyst for the studies without any further purification. Samples treated with the laser are labeled as L-Pd/rGO, while samples after heat treatment are labeled as H-Pd/rGO.

### 2.3 Material Characterization

The powder X-ray diffraction (XRD) patterns of the samples were recorded on a Rigaku Smart Lab SE diffractometer. Raman spectroscopic analysis was conducted using a Horiba Scientific XploRA ONE MICRO-Raman system in Japan, employing a 532 nm laser for excitation. Sample morphologies were examined through scanning electron microscopy (SEM) on a Tescan MAIA3 instrument, which included an energy-dispersive spectrometer (EDS) for elemental analysis. Scanning transmission electron microscopy (STEM) analysis was also carried out using the same instrument. High-resolution transmission electron microscopy (HR-TEM) samples were prepared by drop casting diluted dispersion onto 300 mesh Cu grids and drying overnight. Images and selected area electron diffraction (SAED) patterns were acquired using Tecnai 12 (FEI) and JEM 2100 (JEOL, 200 kV) HR-TEM microscopes. The palladium content of the samples was quantified using inductively coupled plasma-optical emission spectroscopy (ICP-OES) on a Spectro Arcos spectrometer. X-ray photoelectron spectroscopy (XPS) measurements were performed with a Kratos Axis Ultra DLD spectrometer featuring a monochromatic Al Kα X-ray source with an energy of 1486.6 eV. Calibration of high-resolution spectra was achieved using a carbon tape from Ted Pella with a well-established C 1s binding energy of 284.6 eV. Data processing was done via Casa XPS software, utilizing Gaussian–Lorentzian mixtures for peak fitting, except for the sp^2^ C–C component, which was modeled with an asymmetric peak shape. Fourier-transform infrared (FT-IR) spectra of the synthesized catalysts were recorded using a JASCO FT/IR-4700. The N_2_ temperature-programmed desorption (TPD) analysis was done using a Micrometrics AutoChem II chemisorption analyzer coupled with a Hiden analytical HPR-20 QIC benchtop gas analysis system.

### 2.4 Evaluation of Electrocatalytic Nitrate Reduction Reaction

A dual-chamber electrochemical cell with a three-electrode setup was utilized to assess the electrocatalytic nitrate reduction reaction (eNO_3_RR) activity of the synthesized catalysts. The working electrode comprised Teflonized Toray carbon coated with the catalyst; a nickel strip served as the counter electrode, and a Hg/HgO electrode acted as the reference. A pretreated Nafion 115 membrane was used as the separator. The catalyst ink was prepared by ultrasonically dispersing the catalyst in a 1:1 v/v isopropanol and water mixture with 40 wt % Nafion ionomer and then drop-casted onto the Teflonized Toray carbon electrode support (1 cm^2^ area). After drying, the working electrode was formed, with a catalyst loading of ∼2 mg/cm^2^. Electrochemical measurements were conducted in an argon-saturated 1.0 M KOH solution with 0.5 M KNO_3_ using a BioLogic potentiostat/galvanostat (VSP/VMP 3B-20). Potentials were recorded against Hg/HgO and converted to the reversible hydrogen electrode (RHE) scale using the Nernst equation (*E*_RHE_ = *E*_measured_ + *E*_Hg/HgO_ + (0.059 × pH)). An acid trap with 1 mM H_2_SO_4_ was linked to the cell’s outlet to capture the ammonia produced during eNO_3_RR.

### 2.5 Quantification Techniques

#### 2.5.1 Ammonia

The ammonia generated during eNO_3_RR was analyzed by the indophenol blue method as reported elsewhere,^32^ and the total ammonia concentration was reported by adding the ammonia present in both the electrolyte and acid trap. Standard samples were prepared with the known concentration of NH_4_Cl in both 1.0 M KOH and 1 mM H_2_SO_4_ and used to develop a calibration curve for accurate determination of ammonia concentration (Figure S1). An ammonia ion selective electrode (Thermo Scientific Orion, model no: 9512HPBNWP) was further used to reconfirm the indophenol quantification technique. Primarily, the electrode was calibrated with 0.1, 1.0, and 10.0 ppm ammonia samples, and then electrolyte samples were analyzed. The ammonia yield rate (in μg h^–1^ cm^–2^) was calculated using eq 7:
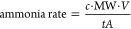
7where *C* is the ammonia concentration (in μM), MW is the molecular weight of ammonia (17.031 g/mol), *V* is the sample volume (in L), *t* is the electrolysis time (in h), and *A* is the electrode active area (in cm^2^). The Faradaic efficiency (FE) was calculated using eq 8:
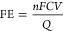
8where *n* is the number of electrons transferred, *F* is the Faraday constant (96 485 C/mol), *C* is the ammonia concentration (in mM), *V* is the volume of the electrolyte (in L), and *Q* is the applied charge (in C).

#### 2.5.2 Nitrate and Nitrite

The concentrations of nitrate and nitrite in the electrolyte were quantified using a UV–visible spectrophotometer, measuring the absorbance at 300 nm for nitrate (NO_3_^–^) and 350 nm for nitrite (NO_2_^–^). Calibration curves for both ions were constructed using known concentrations in 1.0 M KOH and are depicted in Figure S2. The yield rates of nitrate and nitrite, along with their Faradaic efficiencies, were calculated using eqs 7 and 8, incorporating their respective molecular weights.

#### 2.5.3 Hydroxylamine

The concentration of hydroxylamine (NH_2_OH) was determined using a modified colorimetric method adapted from an established protocol reported elsewhere.^30^ A standard calibration curves were developed from known NH_2_OH concentrations in 1.0 M KOH and are depicted in Figure S2. The yield rate and efficiency were calculated by using eqs 7 and 8.

## 3 Results and Discussion

### 3.1 Physiochemical Characterization of the Pd-PdO Doped rGO Catalyst

**Figure 1:**
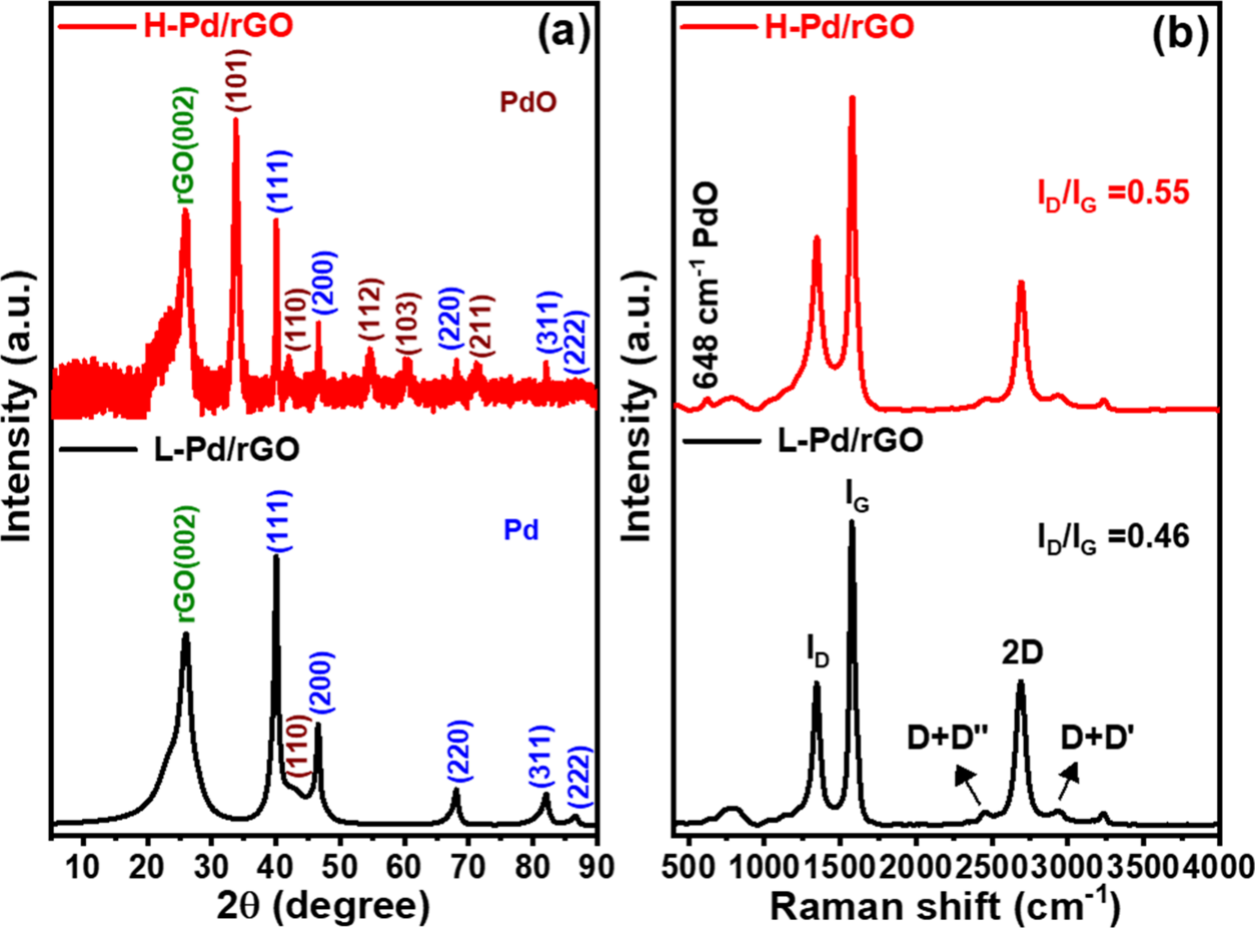
(a) X-ray diffraction pattern and (b) Raman spectra of L-Pd/rGO and H-Pd/rGO (heat treatment at 400 °C).

The Pd-PdO doped rGO composite was synthesized using laser processing followed by heat treatment. The crystal phase and the crystallite structure are analyzed using X-ray diffraction (XRD) and are illustrated in Figure 1a. The XRD pattern of the H-Pd/rGO sample showcases reflections at 2θ values of 33.9, 42.0, 54.6, 60.3, and 71.5°. These correspond to the (101), (110), (112), (103), and (211) planes of tetragonal palladium(II) oxide (PdO, JCPDS 01-075-0584),^33^ along with Pd. This indicates the oxidation of Pd to PdO by atmospheric oxygen. The crystallite size of Pd was calculated as 9.7 nm ((111) plane) using Scherrer’s formula. Following heat treatment, this size increased to 26.4 nm. Additionally, the crystallite size of PdO was calculated to be 32.5 nm. Hence, it is seen that heat treatment under air not only oxidizes Pd but also facilitates its crystallization.^34^ A broad characteristic peak at 26.1° indexed to the (002) plane of rGO was observed in both samples.

Figure 1b explains the changes in the rGO support postheating through Raman spectroscopy analysis. The D vibration band, arising from a breathing mode of A_1g_ symmetry j-point photons,^35^ is observed at 1347.99 cm^–1^ before heat treatment and shifts to 1340.56 cm^–1^ afterward. The G vibration band, associated with first-order scattering of E_2g_ phonons by sp^2^ carbon, appears at 1575.17 cm^–1^ before heating and changes to 1578.78 cm^–1^ postheating. This G band is also influenced by the stretching of C–C bonds in sp^2^ carbon systems.^36^ Additionally, a 2D band is seen at 2699.36 cm^–1^, shifting to a lower wavenumber of 2690.86 cm^–1^ after heat treatment. This shift in the 2D band, which is sensitive to the stacking of graphene layers, confirms the multilayer nature of rGO, as monolayer graphene is typically found at 2679 cm^–1^.^36^ The shift also results from the presence of oxide in PdO, which affects the stacking of graphene layers.^35^ The *I*_D_/*I*_G_ ratio of rGO was determined to be 0.46 before and 0.55 after heat treatment, indicating an increase in sp^2^ carbon restoration and a decrease in the average size of sp^2^ domains upon heating.^37^ A lower intensity in the D band suggests more isolated graphene domains in the heated sample compared to the as-synthesized sample, likely due to the incorporation of oxygen moieties from the air during heating. In addition, the peak at 648 cm^–1^ reassures the formation of PdO during heat treatment.

**Figure 2:**
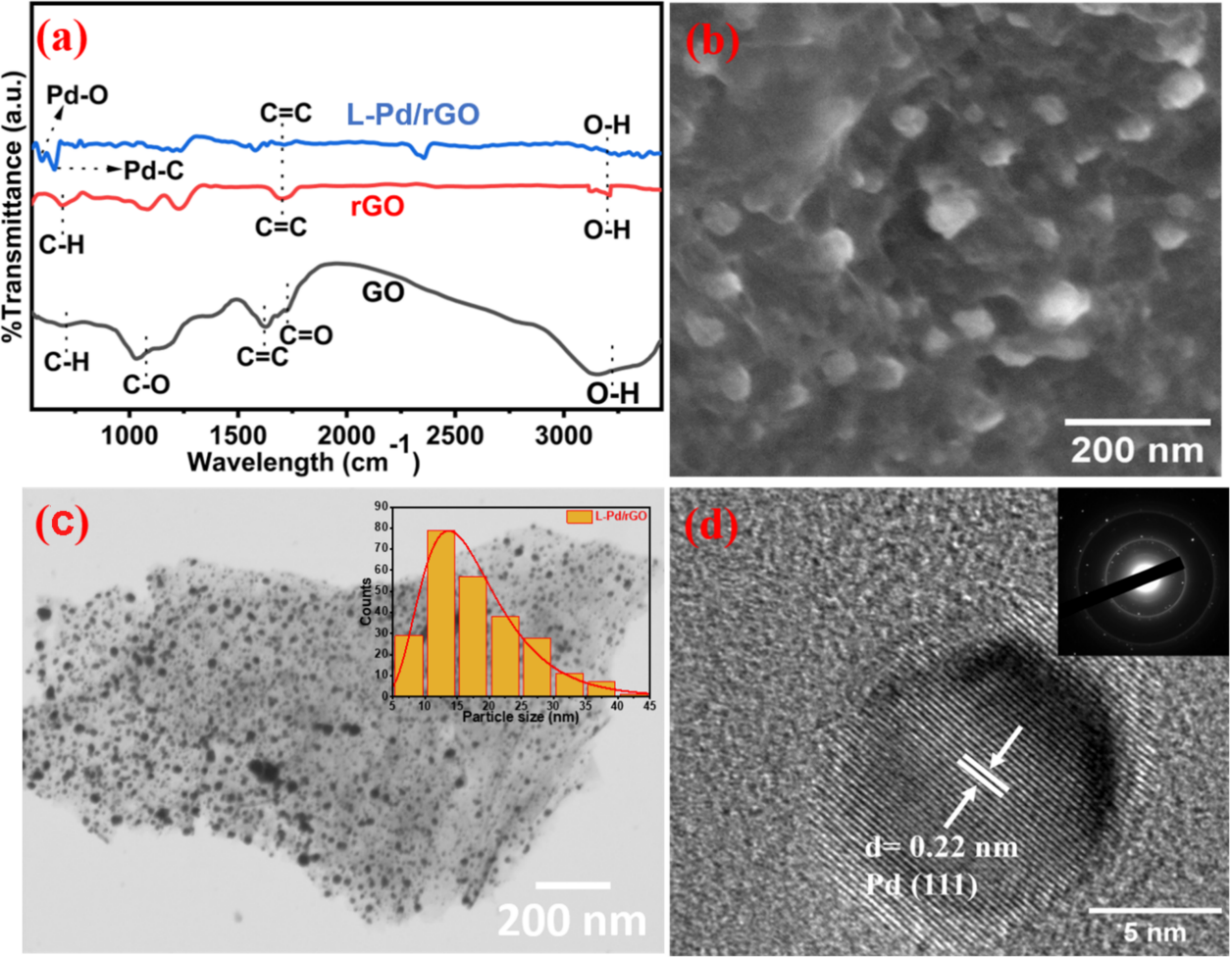
(a) FT-IR spectra of GO, rGO, and L-Pd/rGO. (b) SEM image of the L-Pd/rGO composite. (c) STEM image of L-Pd/rGO. The inset shows the particle size distribution of nanoparticles. (d) HR-TEM image of the L-Pd/rGO composite. The inset shows the SAED pattern.

The chemical composition of L-Pd/rGO is scrutinized using Fourier-transform infrared (FT-IR) spectroscopy. Figure 2a represents the FT-IR spectra of GO, rGO, and L-Pd/rGO (black, red, and blue, respectively). The broad peak at 3275 cm^–1^ designated for OH in GO is diminished on the reduction to rGO. Peaks at 1629 and 1045 cm^–1^ are for aromatic C=C stretching, epoxy C–O stretching, or alkoxy C–O stretching vibration for GO, respectively. Laser-reduction of GO to rGO causes a decrease in the oxygen-containing functionalities, and the C=C band becomes more defined.^38,39^ The weak absorption band present between 680 cm^–1^ corresponds to the bending of the aromatic C–H bond.^40^ Bands formed at 597 and 654 cm^–1^ are due to the vibration of the Pd–O bond and Pd–C bond, respectively.^41,42^

Scanning electron microscopy (SEM) studies reveal a distinctive wrinkled flake shape morphology that resembles the stacked few layers of laser-induced rGO (Figure S3). The thin sheets of graphene account for the monolayers that are readily apparent in the SEM morphology analysis.^39,43^ In the L-Pd/rGO composite, Figure 2b conveys the presence of layers of turbostratic graphene, and highly dispersed Pd and PdO nanoparticles reassure the formation of the nanocomposites. Preferably, the absence of aggregation in Figure 2b corroborates the inviolable adhesion of the nanoparticles to the carbon film.^44^ The uniform distribution of Pd/PdO in both pristine and heat-treated samples is clearly visible in the scanning transmission electron microscope (STEM) images shown in Figures 2c and S5a, respectively. The particle size distribution, calculated from the respective images (16 nm for pristine and 36 nm for heat-treated samples), matches the crystallite sizes determined by XRD. The *d*-spacing of the Pd and PdO lattice fringes was calculated as 0.22 nm for L-Pd/rGO (Figure 2d) and 0.26 nm for H-Pd/rGO (Figure S5), based on high-resolution transmission electron microscopy (HR-TEM) images (Figures S4 and S5). These *d*-spacing values correspond to the (111) plane of Pd and the (101) plane of PdO, aligning with the XRD results. The diffraction rings in the selected area electron diffraction pattern (SAED) (Figures S4d and S5d) confirm the presence of Pd and PdO in the composite. Dark spots dispersed on the background in HR-TEM images indicate high nanoparticle dispersion on rGO in both the L-Pd/rGO and H-Pd/rGO composites. Strong adhesion of nanoparticles to rGO facilitates efficient electron transport, enhancing catalytic activity. Elemental mapping (Figure S6) shows increased oxygen content near Pd in the H-Pd/rGO composite, indicating more PdO formation compared to L-Pd/rGO.^45^ Inductively coupled plasma-optical emission spectroscopy (ICP-OES) analysis results confirm the content of 7 wt % Pd in the composite.

The analysis of the chemical and electronic states of the nanocomposites is performed by using X-ray photoelectron spectroscopy (XPS, Figure S7). XPS is sensitive to the oxidation state of the elements, measuring the binding energy of valence electrons to the surface atom.^46^ The full survey spectrum of the composite, proving the existence of C 1s, O 1s, and Pd 3d, is shown in Figure S7.

**Figure 3:**
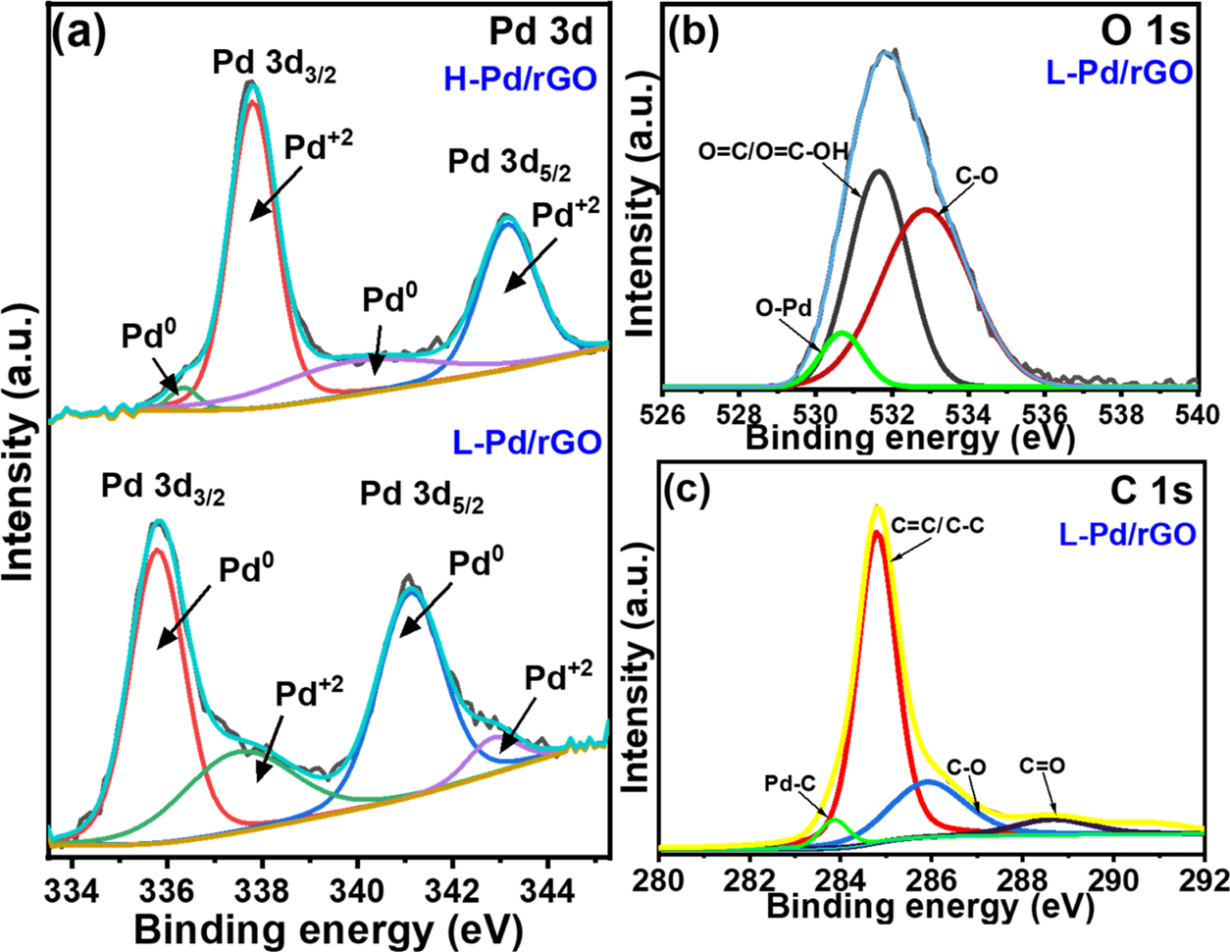
High-resolution XPS spectra of (a) Pd 3d for both L-Pd/rGO and H-Pd/rGO composites and (b) O 1s and (c) C 1s for the L-Pd/rGO composite.

The high-resolution X-ray photoelectron spectrum (XPS) of the Pd 3d spectra is deconvoluted and compared with H-Pd/rGO. Peaks at 335.9 and 341.1 eV in Figure 3a indicate the Pd^0^ oxidation state, while peaks at 337.5 and 334.8 eV correspond to the Pd^2+^ state in the L-Pd/rGO composite.^46−47,48^ These peaks shift to 336.4 and 339.9 eV for Pd^0^ and to 337.8 and 343.2 eV for PdO, indicating increased PdO content. The Pd ratio changes from 0.63:0.37 to 0.27:0.73 post-heat treatment, confirming increased Pd^2+^ and PdO content, consistent with the XRD result. The deconvoluted peaks of O 1s are assigned at 530.6 eV for the Pd–O bond, revealing the PdO phase in the L-Pd/rGO composite.^47,49^ The other O 1s peaks originated from carbon–oxygen bonds at 531.7 and 532.7 eV for the O–C=O and C–O, respectively (Figure 3b).^50,51^Figure S7c compares the O 1s spectra of rGO, L-Pd/rGO, and H-Pd/rGO. In L-Pd/rGO, Pd doping introduces a Pd–O peak at 530.6 eV. In H-Pd/rGO, the peaks shift: Pd–O to 532.3 eV, O–C=O to 537.0 eV, and C–O to 534.0 eV. The PdO peak increases significantly from 13.7% in L-Pd/rGO to 53.3% in H-Pd/rGO, indicating a substantial rise in the PdO content. The C 1s peak of L-Pd/rGO (Figure 3c) can be deconvoluted into four peaks: The majority of the carbon signals detected by XPS (54 atom % from the total composite) originate from C–C and C=C denoted for sp^3^ and sp^2^ carbon at 284.8 eV, confirming the successful conversion of GO to rGO. Still, the presence of C–O and C=O peaks indicates the existence of an oxidized form of carbon in minute quantity (11.6 atom %) from the total composite, Figure 3a.^52,53^ After heat treatment, there is minimal change in the C 1s spectra (Figure S7b) of H-Pd/rGO, with only the Pd–C peak disappearing due to the oxidation of Pd to PdO. This observation is consistent with the Raman spectra.

### 3.2 Electrochemical Nitrate Reduction to Ammonia

**Figure 4:**
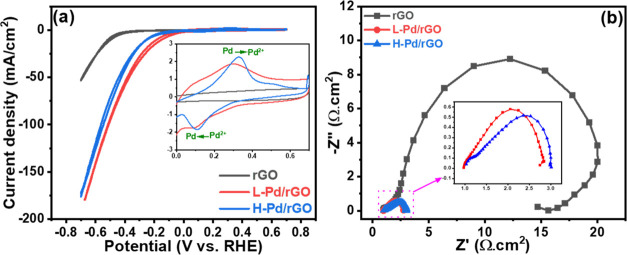
(a) Cyclic voltammogram of rGO, L-Pd/rGO, and H-Pd/rGO coated electrode in 1.0 M KOH containing 0.5 M KNO_3_ solution at a 5 mV/s scan rate. (b) Electrochemical impedance spectroscopy (EIS) results of the same catalysts at −0.4 V vs RHE under the same condition.

The electrocatalytic activity of the L-Pd/rGO and H-Pd/rGO composite catalysts for nitrate reduction was assessed using a three-electrode electrochemical cell equipped with an acid trap. Before conducting electrochemical nitrate reduction experiments, cyclic voltammetry (CV) analysis was performed from 0.7 to −0.7 V vs RHE in 1.0 M KOH with 0.5 M KNO_3_, as illustrated in Figure 4a. The composite exhibited an oxidation peak at 0.31 V for the transition from Pd(0) to Pd(II), with the corresponding reduction peak observed at 0.11 V. In contrast, rGO without Pd doping did not exhibit this redox behavior, confirming the impact of Pd incorporation. Notably, Pd doping of rGO significantly reduced the onset potential for nitrate reduction from −0.43 to −0.01 V, as illustrated in Figure S8.

Furthermore, the influence of Pd doping was also investigated through electrochemical impedance spectroscopy (EIS) in an Ar-saturated 1.0 M KOH solution containing 0.5 M KNO_3_ at −0.4 V, presented in Figure 4b. Post-Pd doping, the charge transfer resistance for nitrate reduction markedly decreased from 19.3 to 2.0 Ω on the same electrode. Additionally, following heat treatment, the charge transfer resistance slightly increased to 2.3 Ω, which was attributed to the formation of PdO, which inherently possesses higher resistance. The electrochemical accessible surface area of the rGO, L-Pd/rGO, and H-Pd/rGO electrocatalysts was performed at different scan rates of CV within a non-Faradaic potential range in 1.0 M KOH with a 0.5 M KNO_3_ electrolyte (Figures S9 and S10) and is tabulated in Table 1.

**Table 1:** Electrochemical Double-Layer Capacitance (Cdl) and Electrochemical Surface Area (ECSA) for rGO and Pd-PdO/rGO Electrodes in 1.0 M KOH Containing 0.5 M KNO_3_ Solution

sample	Cdl (mF/cm^2^)	ECSA (cm^2^/g)
L-rGO	0.00969	180.78
H-rGO	0.00456	100.88
L-Pd/rGO	0.03055	454.61
H-Pd/rGO	0.02263	340.81

**Figure 5:**
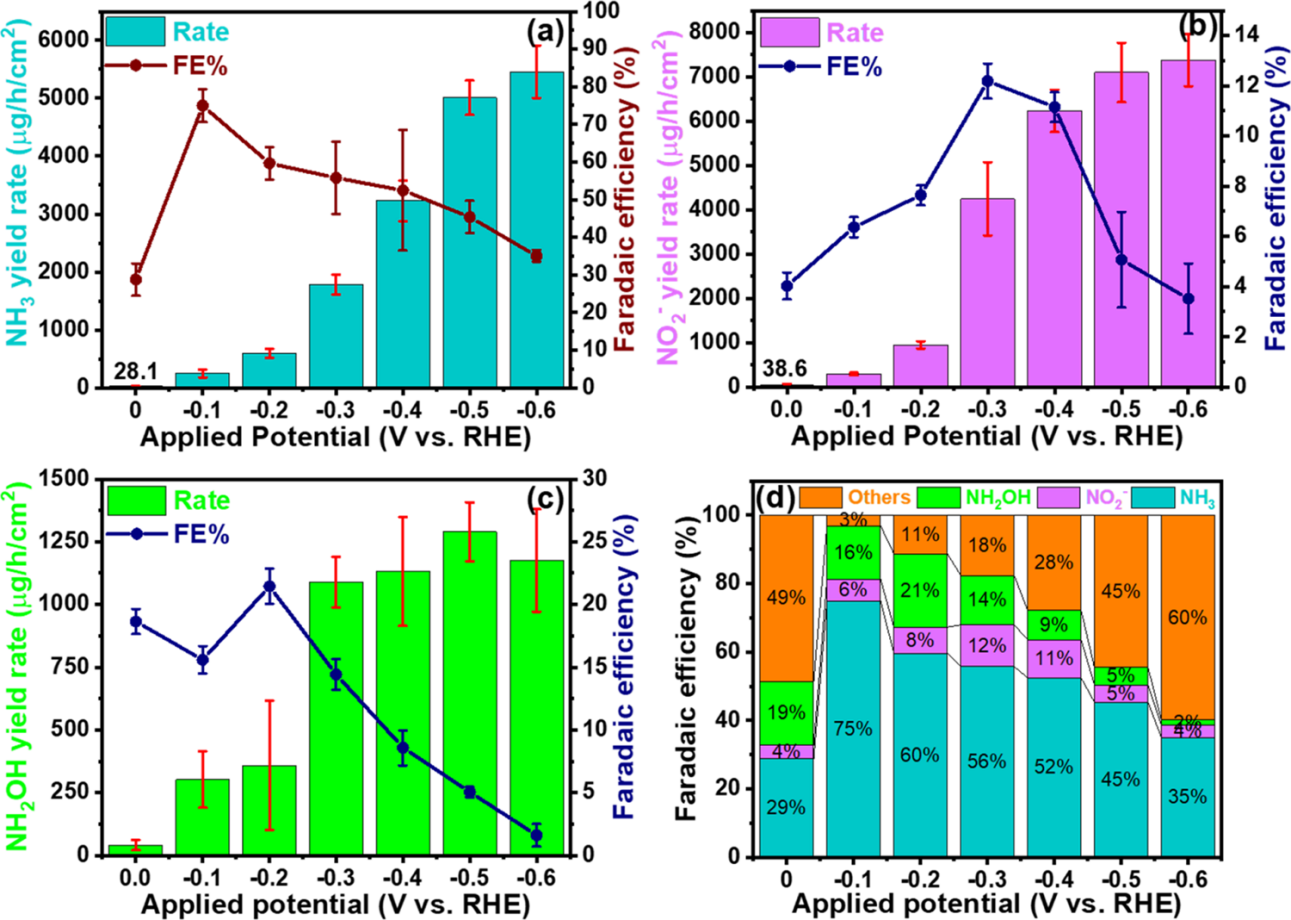
(a) Ammonia, (b) nitrite, (c) hydroxylamine yield rate and corresponding faradaic efficiency of the H-Pd/rGO catalyst at selected applied potentials and (d) Faradaic efficiency distribution of the H-Pd/rGO sample at different applied potentials.

Table 1 presents the electrochemical double-layer capacitance (Cdl) and the equivalent electrochemical surface area (ECSA) of reduced graphene oxide (rGO) and palladium/palladium oxide-decorated rGO (Pd-PdO/rGO), both in their original state and after 2 h of heat treatment at 400 °C in air. The data depicts a significant decrease in ECSA of both materials post heating. This reduction is likely due to graphene layer agglomeration or restacking, caused by the removal of surface functional groups at high temperatures that initially prevented restacking. The Raman spectra in Figure S11 show a decrease in the *I*_D_/*I*_G_ ratio from 1.13 to 0.46, explaining the 44% ECSA reduction in rGO vs 25% in Pd-doped rGO, highlighting Pd-PdO’s role in enhancing and retaining ECSA after heat treatment. The EIS on open circuit voltage (0.07 V) also (Figure S8) shows a similar decrease of charge transfer resistance after heat treatment (H-Pd/rGO < L-Pd/rGO < H-rGO < L-rGO). To further validate the catalyst’s performance, chronoamperometric (CA) electrolysis was conducted for 1 h at seven selected potentials ranging from 0.0 to −0.6 V, as shown in Figure S8. The ammonia concentration produced in both the electrolyte and the trap was measured using the indophenol blue method and analyzed with a UV–visible spectrophotometer. The UV–visible spectra of the electrolyte samples are presented in Figure S12, while the calculated ammonia yield rate is depicted in Figure 5a. At a potential of 0.0 V vs RHE, the ammonia yield rate was measured as 28.1 ± 2.1 μg/h/cm^2^ with a Faradaic efficiency (FE) of 28.8 ± 4.3%. This yield rate exhibited a nearly linear increase, reaching 246.1 ± 68.5 μg/h/cm^2^ at −0.1 V and further rising to 5008.9 ± 29.8 μg/h/cm^2^ at −0.5 V, beyond which it plateaued. Concurrently, the FE increased to 74.9 ± 4.4% at −0.1 V, slightly decreasing to 52.4 ± 15.9% up to −0.4 V before beginning to decline sharply. A similar pattern of linear growth up to −0.5 V followed by saturation was also observed in the nitrite yield rate, as shown in Figure 5b. The maximum yield rate for nitrite reached 7381.1 ± 59.5 μg/h/cm^2^ at −0.6 V, slightly exceeding the yield at −0.5 V. The FE for nitrite production increased from 6.4 ± 0.3% at −0.1 V to 12.2 ± 0.7% at −0.3 V and then decreased at higher applied potentials. Regarding hydroxylamine production, the FE reached its maximum of 21.4 ± 1.4% at −0.2 V, as indicated in Figure 5c, and diminished at higher potentials. The highest hydroxylamine yield rate of 1289.3 ± 118.1 μg/h/cm^2^ was achieved at −0.5 V.

Figure 5d presents the distribution of the FE for each product across varying applied potentials. At a lower potential of −0.1 V vs RHE, only 3.0 ± 2.2% of the efficiency was lost to parasitic reactions, primarily due to the hydrogen evolution reaction (HER), with the remainder distributed as 74.9 ± 4.4% for ammonia and 6.4 ± 0.3 and 15.6 ± 1.1% for nitrite and hydroxylamine, respectively. At higher overpotentials, such as −0.6 V, a significant shift in efficiency toward the HER was observed, accounting for 59.8 ± 4.2% of the total. To minimize losses to the parasitic HER with a relatively higher ammonia yield rate, an applied potential of −0.4 V was identified as optimal. This potential facilitated the production of ammonia at a rate of 3226.6 ± 35.3 μg/h/cm^2^ with an FE of 52.4 ± 15.9%, while 11.2 ± 0.6 and 8.6 ± 1.4% of the efficiency resulted in the production of nitrite and hydroxylamine, respectively. Furthermore, the performance of Pd-PdO doped reduced graphene oxide (rGO) was benchmarked against that of undoped rGO (refer to Figure S13). The results demonstrated that the ammonia production rate of Pd-PdO doped rGO at a potential of −0.5 V was 5.51 times higher than that of bare rGO. This increase is attributed to the doping of Pd-PdO and the 3.9-fold greater electrochemical surface area (ECSA) after doping.

### 3.3 Stability of the Catalyst System

**Figure 6:**
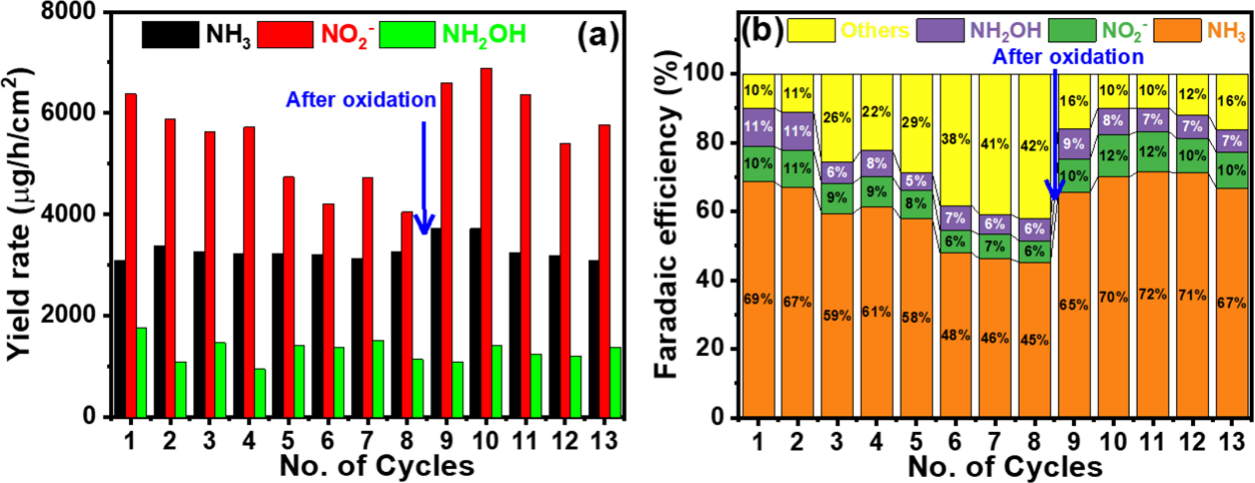
(a) Ammonia, nitrite, and hydroxylamine yield rate and (b) the corresponding faradaic efficiency of the H-Pd/rGO catalyst upon consecutive eNO_3_RR cycles (1 h CA) at −0.4 V vs RHE in Ar-saturated 1.0 M KOH containing 0.5 M KNO_3_ solution.

The stability of the H-Pd/rGO composite catalyst system was evaluated in a 1.0 M KOH solution containing 0.5 M KNO_3_, performing nitrate reduction in repeated cycles. Each cycle consisted of 1 h of CA at −0.4 V vs RHE, with both the electrolyte and acid trap being replaced between cycles to avoid large changes in the concentrations of nitrate, nitrite, hydroxylamine, and ammonia, which might affect the reduction rate. The yield rates of ammonia, nitrite, and hydroxylamine, along with their respective FEs, are documented in Figure 6a,b. In the first cycle, yield rates of 3096.9 μg/h/cm^2^ for ammonia, 6375.0 μg/h/cm^2^ for nitrite, and 1152.5 μg/h/cm^2^ for hydroxylamine were measured. The yield rate of ammonia increased slightly to 3376.6 μg/h/cm^2^ in the subsequent cycle and then began to decrease gradually until the eighth cycle. Conversely, the yield rate of nitrite decreased from 6375.0 μg/h/cm^2^ in the first cycle to 4048.1 μg/h/cm^2^ by the eighth cycle, a trend that was similarly observed for hydroxylamine. The distribution of FE for each product, as illustrated in Figure 6b, indicates a shift in efficiency toward the HER away from ammonia, nitrite, and hydroxylamine production. X-ray diffraction analysis of the electrode (shown in Figure S14) revealed the disappearance of some reflections at 2θ values of 41.8, 60.6, 62.2, and 70.8° corresponding to the (110), (103), (200), and (202) planes of PdO, along with a parallel enhancement of characteristic Pd planes. This suggests that some of the oxides in the H-Pd/rGO composite were reduced to Pd, leading to a decrease in the catalyst system’s activity. This change also increased the defects in the rGO, visible from the increased *I*_D_/*I*_G_ ratio to 0.95 from 0.45, as mentioned in the Raman spectra in Figure S14.

The diminished activity of the H-Pd/rGO composite system was successfully restored by reoxidizing the reduced Pd back to PdO. Following methodologies from the existing literature,^54^ this oxidation process was performed electrochemically in a 1.0 M KOH solution at a potential of 0.95 V vs RHE for one hour. With the electrode reoxidized, the electrochemical nitrate reduction reaction (eNO_3_RR) was sustained for up to 5 additional cycles. As seen in Figure 6a,b, the yield rates of ammonia and nitrite were restored to 3720.6 and 6592.9 μg/h/cm^2^, respectively. However, the yield rate for hydroxylamine remained unchanged. The faradaic efficiency for the products also improved, rising to 65.5% for ammonia (up from 45.2%), 9.7% for nitrite (up from 6.4%), and 8.8% for hydroxylamine (up from 6.4%). This reactivation strategy underscores the potential for the long-term, cyclic use of the H-Pd/rGO composite in nitrate-to-ammonia electrochemical conversion processes. The ammonia concentrations after stability experiments were also validated by the ammonia ion selective electrode method (see Table S1), and a 4.6% average deviation from UV–visible measurement was calculated.

## 4 Mechanism of Nitrate Reduction

**Figure 7:**
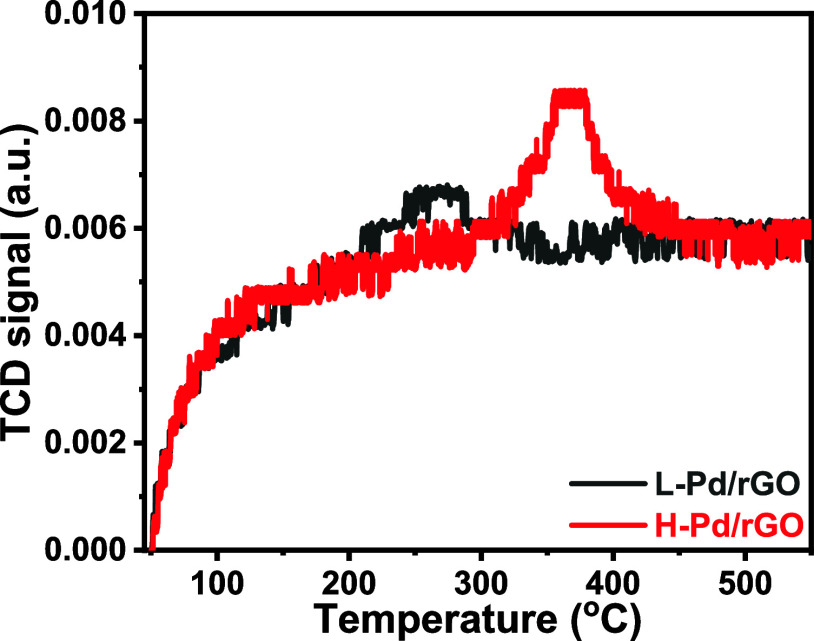
N_2_-temperature-programmed desorption profiles of L-Pd/rGO and H-Pd/rGO composites.

Stability studies indicated a partial reduction of PdO during the electrochemical nitrate reduction reaction, suggesting the direct involvement of PdO in the reduction mechanism. Hence, temperature-programmed desorption analysis using nitrogen (N_2_ TPD) measurements was conducted on both L-Pd/rGO and H-Pd/rGO samples. These analyses were performed up to 550 °C at a heating rate of 2 °C/min, employing helium as the carrier gas; results are depicted in Figure 7. Key indicators, such as desorption temperature and desorption peak intensity, were evaluated to deduce the relative number of active catalyst sites.

In L-Pd/rGO, nitrogen desorption was observed at around 260 °C, whereas for the heat-treated samples, this desorption peak shifted to a higher temperature, centered at 370 °C. This shift to higher temperatures indicates a stronger nitrogen adsorption^55^ on PdO compared to Pd. Further analysis using online mass spectrometry (shown in Figure S15) revealed that nitrogen was desorbed as nitrous oxide (N_2_O, *m*/*z* = 44) for the H-Pd/rGO, in contrast to the exclusive desorption of N_2_ for the pristine sample. Also, the N_2_ adsorption capacity was increased to 29.5 cm^3^/g in H-Pd/rGO from 14.9 cm^3^/g. XRD analysis, as shown in Figure S16, also confirmed this reduction of PdO to Pd, corroborating the role of lattice oxygen in the reduction mechanism. The Raman spectral analysis of the samples following N_2_ TPD (see Figure S17) indicates the formation of defects resulting from oxygen loss. The *I*_D_/*I*_G_ ratio of H-Pd/rGO rose 1.64 times, highlighting the effect of oxygen removal. The background N_2_-TPD of rGO (without Pd doping) shown (Figure S18) shows no desorption signal under the same condition, revealing that the activity comes from PdO.

Our previous study in electrochemical nitrogen reduction using the RuO_2_ catalyst,^55^ highlighted the critical role of oxygen vacancies (V_o_) metallic Ru interactions in enhancing the electrocatalytic nitrogen reduction to ammonia under ambient conditions. Specifically, the presence of V_o_ sites facilitates the adsorption of nitrogen molecules and provides additional active sites for the reduction process alongside the metallic Ru phase. Adopting this understanding, the eNO_3_RR mechanism for Pd-PdO/rGO may be described as shown in eqs 9–13. Initially, the lattice oxygen in PdO sites on the rGO is partially reduced, creating Pd^0^ sites while also generating vacancies on the oxide surface. These vacancies act as active sites for nitrate adsorption. Once adsorbed, nitrate undergoes a two-electron reduction to nitrite to form an *NO intermediate. Subsequently, this intermediate is protonated at the Pd site, which has a high affinity for hydrogen adsorption.^56^ This affinity has been theoretically and experimentally validated in Pd-based catalysts for hydrogen storage reactions.^57^ The hydrogen adsorbed on Pd then spills over^58^ to the adsorbed nitrate, leading to the formation of NH_2_OH and ultimately ammonia. The presence of vacancies (by the partial reduction of PdO to Pd during eNO_3_RR) on PdO may further facilitate this process by providing additional adsorption and activation sites, thus enhancing the overall reduction efficiency and selectivity toward ammonia formation. Moreover, these active sites were being depleted upon duration and can be created back by electrooxidation, as mentioned in the previous section.
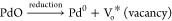
9
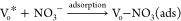
10
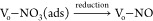
11
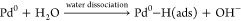
12
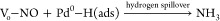
13

Figure S19 compares the Frost–Ebsworth diagram^59^ of electrochemical nitrate reduction pathways, starting from nitrate (NO_3_^–^). The diagram presents two routes: the preferred ammonia route (blue) in alkaline pH and the nitrogen route (green) in acidic pH. In the ammonia route, nitrate is reduced through intermediates such as nitrite (NO_2_^–^), nitric oxide (NO), and hydroxylamine (NH_2_OH), and finally to ammonia (NH_3_), highlighting a favorable reduction process in our alkaline conditions. The nitrogen route also starts with nitrate but proceeds through nitrous oxide (N_2_O), nitrogen gas (N_2_), and other intermediates, representing different oxidation states that are possible under acidic conditions.

The Gibbs free energy change (Δ*G*°) for the electrochemical nitrate reduction reaction (eNO_3_RR) involves multiple steps, each with distinct energetics. The process begins with the adsorption of nitrate (NO_3_^–^) on the catalyst surface. The first reduction step converts NO_3_^–^ to nitrite (NO_2_^–^) with an energy increase of 24.3 kJ/mol. The second reduction to nitric oxide (NO) required 18.6 kJ/mol. The subsequent reduction of NO to hydroxylamine (NH_2_OH) is highly endothermic with a Δ*G*° of 186.6 kJ/mol. Finally, reducing NH_2_OH to ammonia (NH_3_) is an exothermic reaction with a Δ*G*° of −137.3 kJ/mol.

These values highlight the energy demands and releases at each step, which are comparatively lower than the direct nitrogen reduction reaction.^60^ This comparison underscores the efficiency of the ammonia route in nitrate reduction, making it a more favorable pathway for practical applications. Moreover, online mass spectrometry analysis of the system, as presented in Figure S20, demonstrates that no other nitrogen species, such as N_2_O, NO, N_2_, or N_2_O_5_, were generated during the process, aside from the hydrogen evolution reaction. This evidence underlines the selectivity of the catalytic system and its potential for long-term applications in nitrate reduction.

## 5 Conclusions

In conclusion, the electrochemical reduction of nitrate represents a dual-benefit approach, offering a sustainable pathway for green ammonia production while addressing environmental concerns related to nitrate pollution. To improve selectivity toward complete nitrate-to-ammonia conversion, we developed a laser-processed Pd-PdO/rGO composite catalyst. This catalyst achieved remarkable performance, with an ammonia production rate of 5456.4 ± 453.4 μg/h/cm^2^ at −0.6 V vs RHE and additional yields of 7381.1 ± 594.7 μg/h/cm^2^ for nitrite and 1175.3 ± 204.4 μg/h/cm^2^ for hydroxylamine. At a potential of −0.1 V, the catalyst showed minimal hydrogen evolution (3.0 ± 2.2%), favoring ammonia with a selectivity of 74.9 ± 4.4% and allocating the balance to nitrite (6.4 ± 0.3%) and hydroxylamine (15.6 ± 1.1%). Furthermore, the catalyst’s performance and stability were efficiently maintained up to 13 cycles of eNO_3_RR at −0.4 V through the electrooxidation of Pd. This catalyst strategy markedly boosts the electrochemical nitrate reduction efficiency, showcasing a promising catalyst system for environmentally friendly ammonia synthesis.

## Supporting Information Available

UV–visible spectra of the electrolyte, trap, and standard samples; SEM images of rGO; HR-TEM images of L-Pd/rGO and H-Pd/rGO; XPS survey spectrum of rGO, L-Pd/rGO, and H-Pd/rGO; LSV and CA plots of the H-Pd/rGO composite; XRD and Raman spectra of H-Pd/rGO after N_2_ TPD; online mass spectra of H-Pd/rGO during N_2_-TPD and eNO_3_RR; N_2_-TPD datum of rGO; ammonia concentration comparison with ISE; and performance comparison with other catalyst systems (PDF)
