# Supplementary material for: Laser-Induced Pd-PdO/rGO Catalysts for Enhanced Electrocatalytic Conversion of Nitrate into Ammonia
Source: ACS Appl Mater Interfaces. 2024 Jul 4;16(28):36433–43. doi: 10.1021/acsami.4c06378 (PMC11261573; doi:10.1021/acsami.4c06378)
Supplement: Supplementary file 1 — am4c06378_si_001.pdf [file am4c06378_si_001.pdf]

Supporting Information

Laser-induced Pd-PdO/rGO Catalysts for Enhanced Electrocatalytic Conversion of Nitrate into Ammonia

James Ebenezer,[a‡] Aneena Lal,[a‡] Parthiban Velayudham,[a] Arie Borenstein,[a] and Alex Schechter[a,b*]*

[a] Department of Chemical Sciences, Ariel University, Ariel 40 700, Israel.

[b] Research and Development Centre for Renewable Energy, New Technology Centre, University of West Bohemia, 301 00 Pilsen, Czech Republic.

[*] Email: salex@ariel.ac.il, arieb@ariel.ac.il; Tel.: +972 39371470; fax: +972 547740254

[‡]JE and AL contributed equally to this work

Contents

Figure S1. UV-visible spectra of ammonia samples in (a) 1.0 M KOH, (c) 1.0 mM H_2SO_4 solution using Indophenol blue method, and (b and d) their respective linear fitting.

Figure S2. Calibration plots of (a) nitrate, (b) nitrite, and (c) hydroxylamine standard samples in 1.0 M KOH solution.

Figure S3. Scanning electron microscopic (SEM) images of rGO in different magnifications.

Figure S4. (a)-(c) High resolution transmission electron microscopic (HR-TEM) images of L-Pd/rGO at different scales, and (d) selected area electron diffraction pattern (SAED) of L-Pd/rGO.

Figure S5. (a) Scanning transmission electron microscopic (STEM) image (insert shows particle distribution of nanoparticles); (b), (c) High-resolution transmission electron microscopic (HR-TEM) images of H-Pd/rGO at different scales, (d) SAED pattern of H-Pd/rGO.

Figure S6. Energy dispersive X-ray spectrometry (EDS) elemental mapping of L-Pd/rGO (a) palladium, (b) oxygen, (c) carbon, and H-Pd/rGO (d) palladium, (e) oxygen, and (f) carbon.

Figure S7. (a) X-ray photoelectron spectroscopic (XPS) survey spectra, (b) high-resolution C 1s, and (c) high-resolution O 1s spectra of rGO, L-Pd/rGO, and H-Pd/rGO composite catalysts.

Figure S8. (a) linear sweep voltammogram (scan rate 5 mV/s), (b) electrochemical impedance spectroscopy (EIS) results, and (c) chronoamperometric plot of H-Pd/rGO composite catalyst in Ar saturated 1.0 M KOH containing 0.5 M KNO_3 solution. The small peak at 0.11 V vs. RHE is PdO reduction.

Figure S9. Cyclic voltammograms of (a) L-rGO, (c) H-rGO measured at different scan rates in Ar-saturated 1.0 M KOH containing 0.5 M KNO_3; and linear plot of capacitive current of (b) L-rGO, (d) H-rGO measured as function of CV scan rate.

Figure S10. Cyclic voltammograms of (a) L-Pd/rGO, (c) H-Pd/rGO measured at different scan rates in Ar-saturated 1.0 M KOH containing 0.5 M KNO_3; and linear plot of capacitive current of (b) L-Pd/rGO, (d) H-Pd/rGO measured as function of CV scan rate.

Figure S11. Raman spectra of L-rGO and H-rGO (at 400 □ in air for 2 hrs).

Figure S12. UV-visible spectra of (a) electrolyte (diluted 60 times) in indophenol ammonia quantification, (b) trap (diluted 25 times) in indophenol ammonia quantification, (c) electrolyte in ferricyanide hydroxylamine quantification, and (d) electrolyte in nitrite quantification.

Figure S13. (a) ammonia/nitrite/hydroxylamine yield rate, and (b) faradaic efficiency comparison of H-rGO and H-Pd/rGO composite catalyst at -0.5 V vs. RHE in Ar saturated 1.0 M KOH containing 0.5 M KNO_3 solution.

Figure S14. (a) X-ray diffraction pattern, and (b) Raman spectra of L-Pd/rGO coated electrode after 8 hours of eNO_3RR cycles at -0.4 V vs. RHE.

Figure S15. (a) Online mass spectrum recorded during the degassing at 200 °C, nitrogen purging at 50 °C and N_2-TPD experiment of H-Pd/rGO, and (b) magnified view of nitrogen and nitrous oxide gas in the region of N_2 desorption.

Figure S16. X-ray diffraction pattern of (a) L-Pd/rGO, and (b) H-Pd/rGO composite catalysts after N_2 TPD analysis.

Figure S17. Raman spectra of (a) L-Pd/rGO, and (b) H-Pd/rGO composite catalysts after N_2 TPD analysis.

Figure S18. N_2-Temperature programmed desorption profile of H-rGO (at 400 □ in air for 2 hrs).

Figure S19. (a) Frost-Ebsworth diagram of nitrogen species at pH 0.0 (green) and (blue) pH 14.0.

Figure S20. Online mass spectra recorded at -0.4 V vs. RHE for H-Pd/rGO composite catalyst-coated electrode in Ar-saturated 1.0 M KOH solution containing 0.5 M KNO_3.

Table S1. Comparison of ammonia concentration analyzed by ammonia ion selective electrode and Indophenol method.

Table S2. Comparison of H-Pd/rGO composite catalyst performance with that of previously reported catalysts.

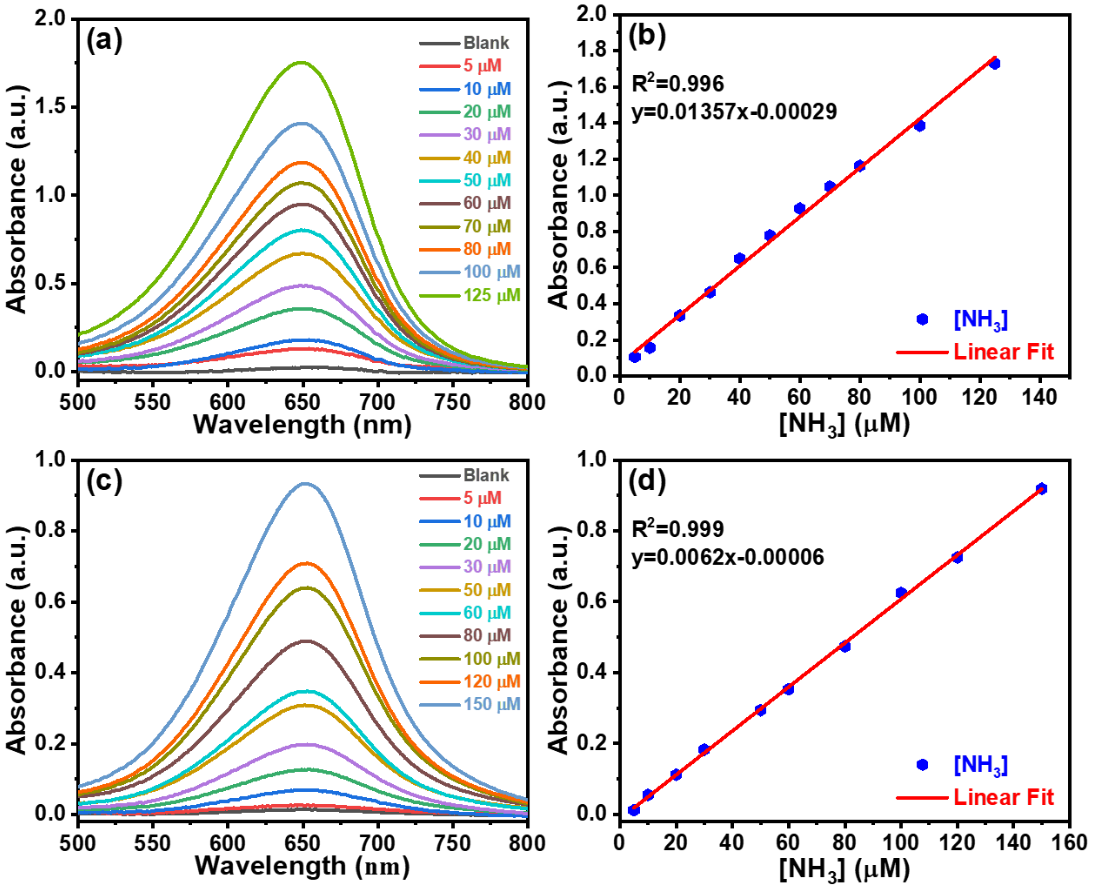

Figure S1. UV-visible spectra of ammonia samples in (a) 1.0 M KOH, (c) 1.0 mM H_2SO_4 solution using the Indophenol blue method, and (b and d) their respective linear fitting.

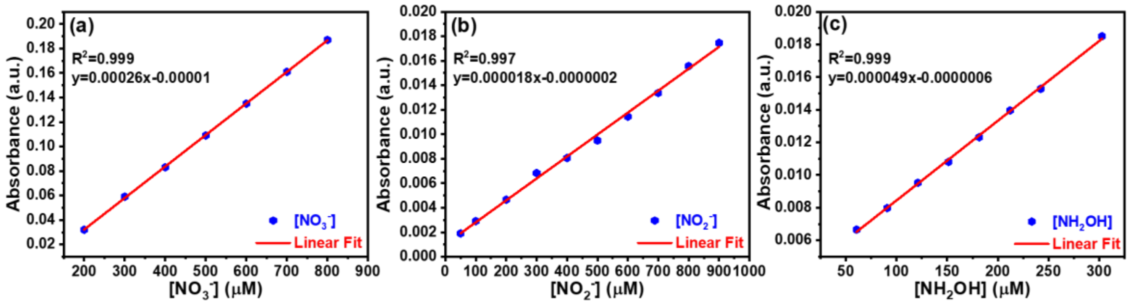

Figure S2. Calibration plots of (a) nitrate, (b) nitrite, and (c) hydroxylamine standard samples in 1.0 M KOH solution.

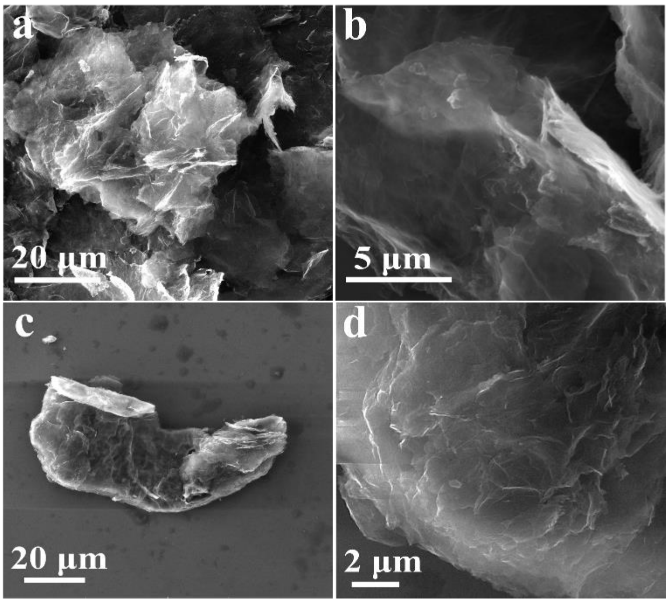

Figure S3. (a) Scanning electron microscopic (SEM) images of rGO in different magnifications.

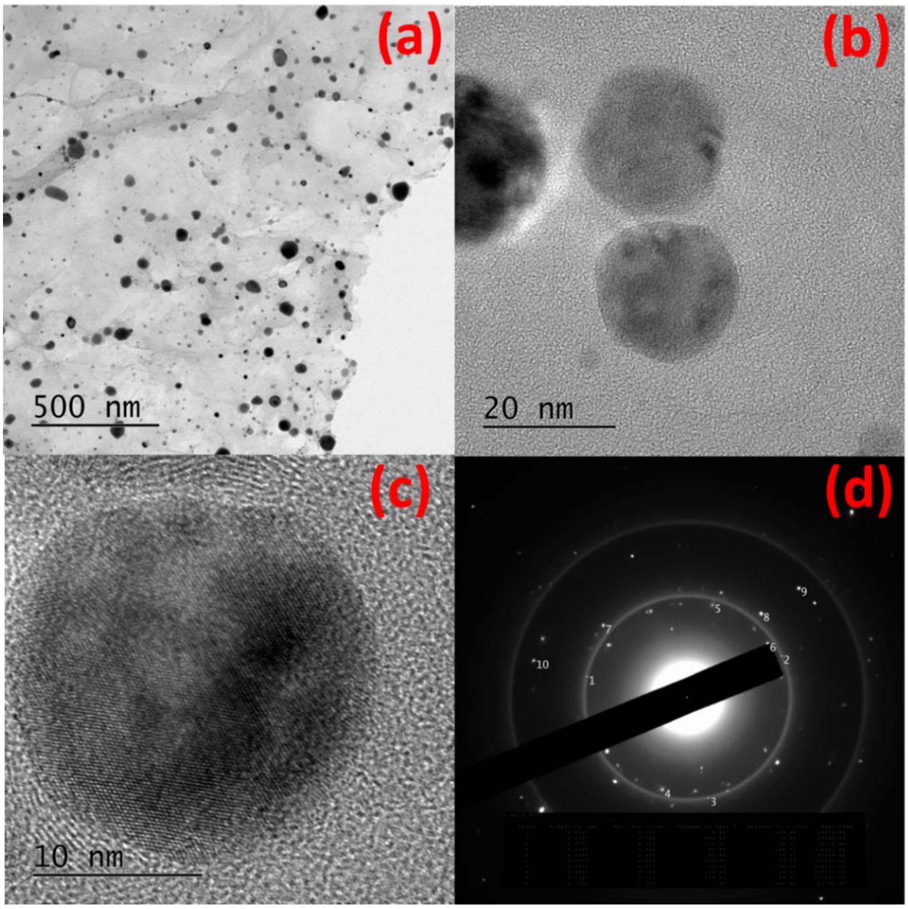

Figure S4. (a)-(c) High resolution transmission electron microscopic (HR-TEM) images of L-Pd/rGO at different scales, and (d) selected area electron diffraction pattern (SAED) of L-Pd/rGO.

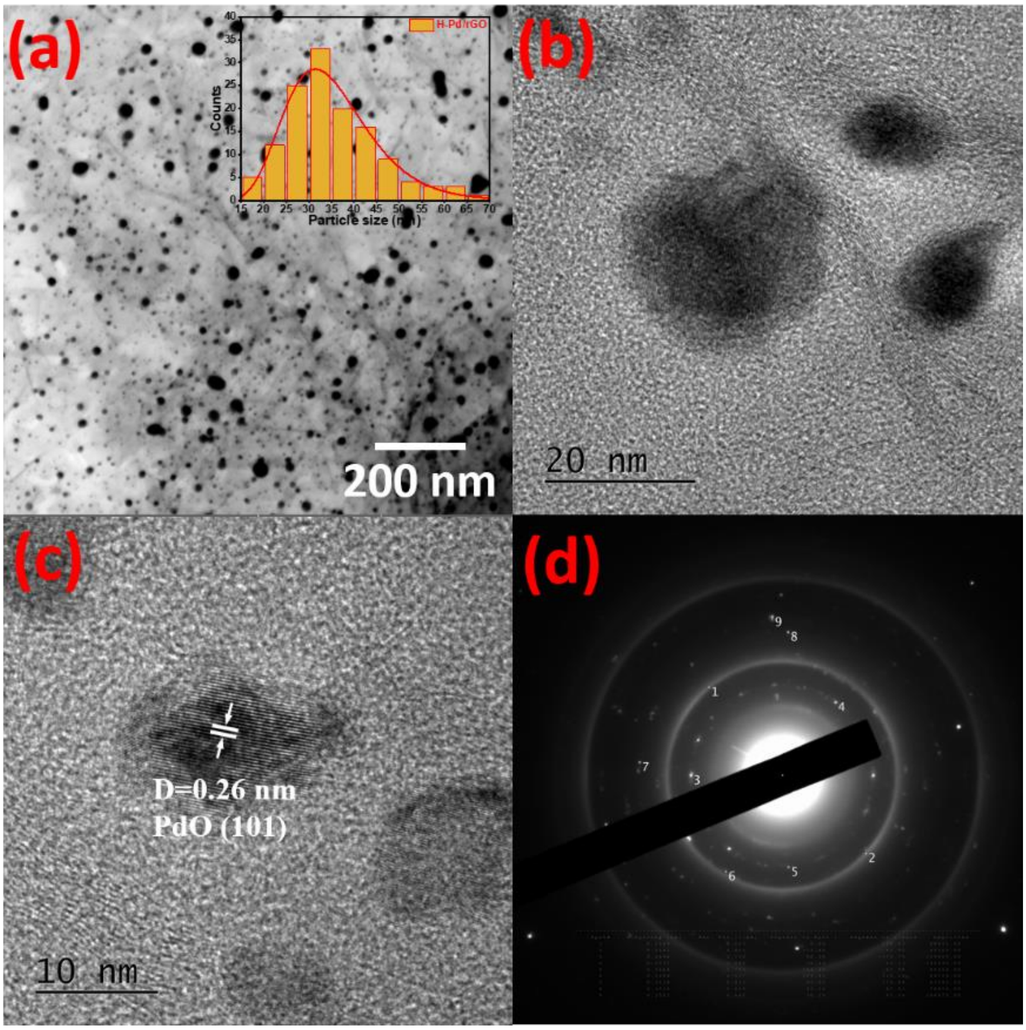

Figure S5. (a) Scanning transmission electron microscopic (STEM) image (insert shows particle distribution of nanoparticles); (b), (c) High-resolution transmission electron microscopic (HR-TEM) images of H-Pd/rGO at different scales, (d) SAED pattern of H-Pd/rGO.

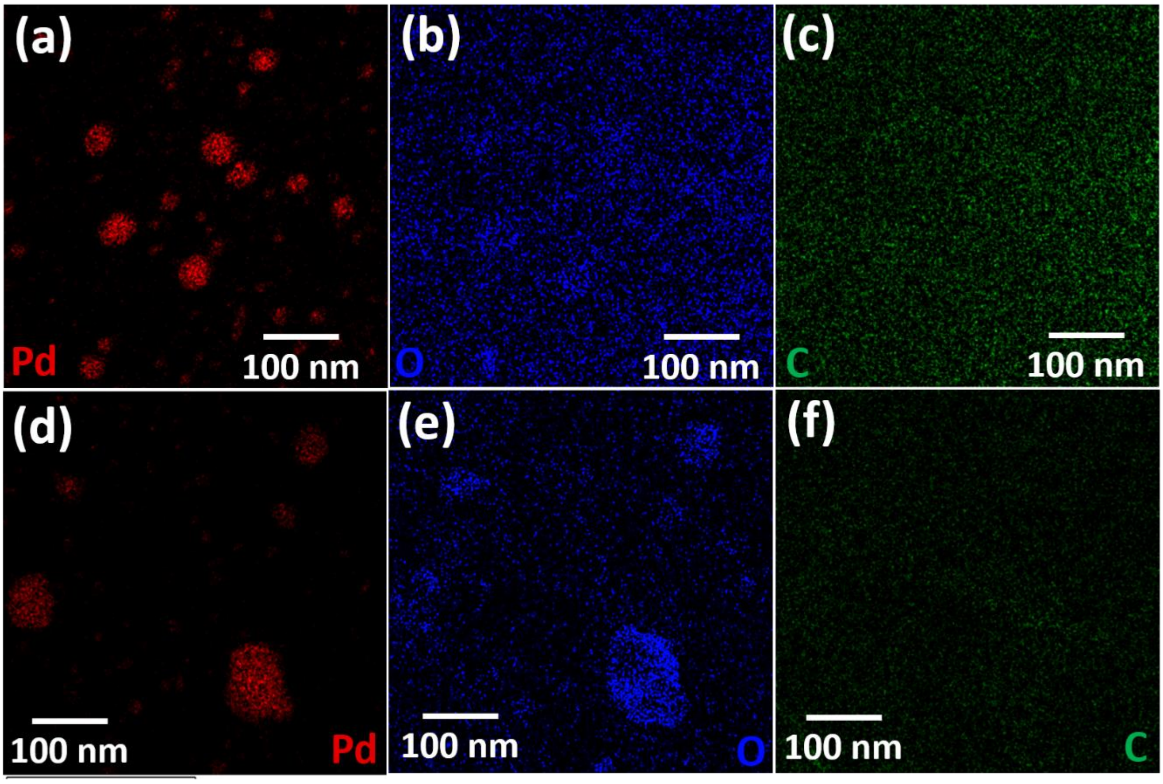

Figure S6. Energy dispersive X-ray spectrometry (EDS) elemental mapping of L-Pd/rGO (a) palladium, (b) oxygen, (c) carbon, and H-Pd/rGO (d) palladium, (e) oxygen, and (f) carbon.

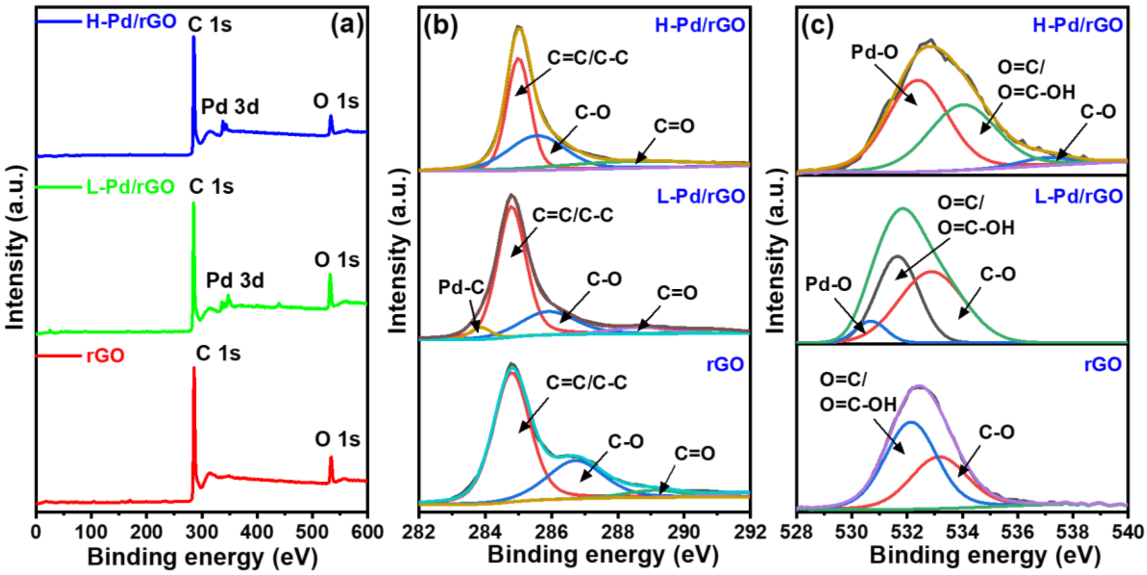

Figure S7. (a) X-ray photoelectron spectroscopic (XPS) survey spectra, (b) high-resolution C 1s, and (c) high-resolution O 1s spectra of rGO, L-Pd/rGO, and H-Pd/rGO composite catalysts.

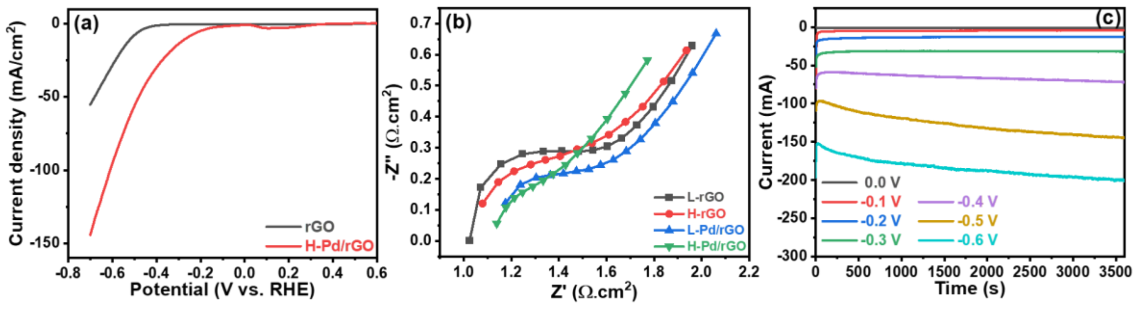

Figure S8. (a) linear sweep voltammogram (scan rate 5 mV/s), (b) electrochemical impedance spectroscopy results at open circuit potential, and (c) chronoamperometric plot of H-Pd/rGO composite catalyst in Ar saturated 1.0 M KOH containing 0.5 M KNO_3 solution. The small peak at 0.11 V vs. RHE is PdO reduction.

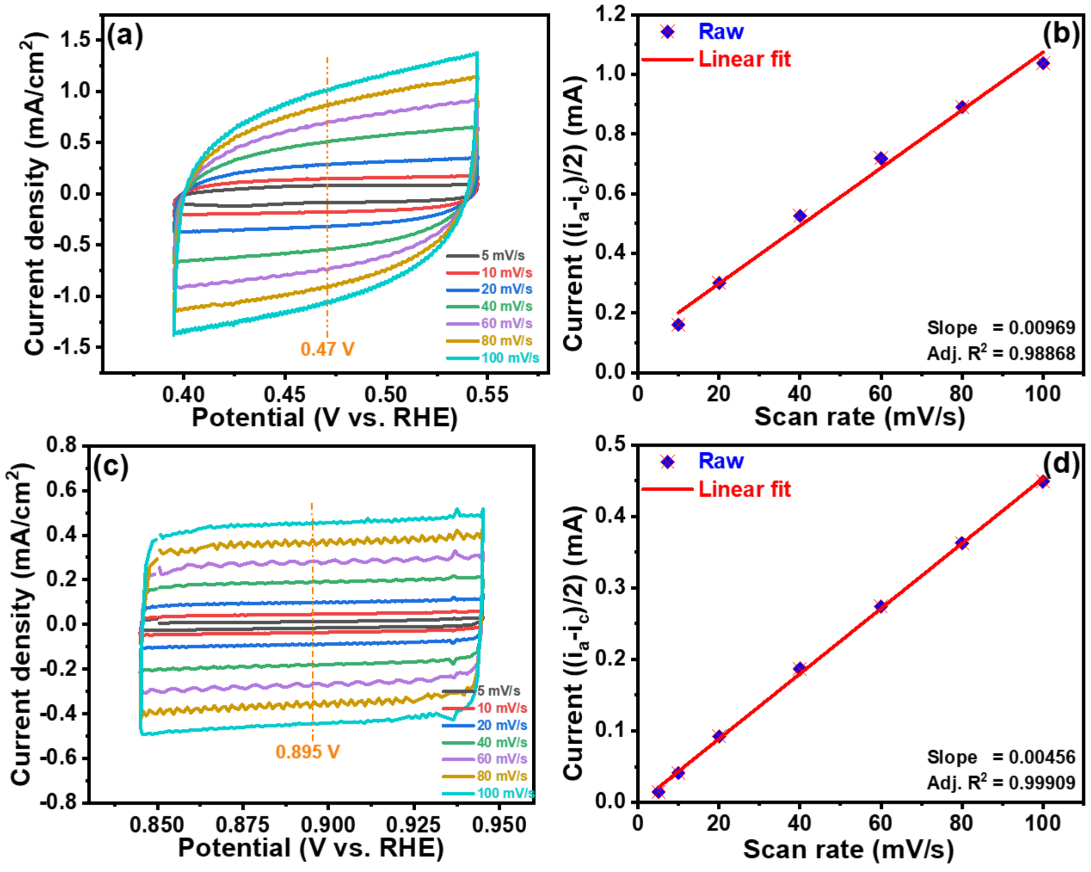

Figure S9. Cyclic voltammograms of (a) L-rGO, (c) H-rGO measured at different scan rates

in Ar-saturated 1.0 M KOH containing 0.5 M KNO_3; and linear plot of capacitive current of (b) L-rGO, (d) H-rGO measured as function of CV scan rate.

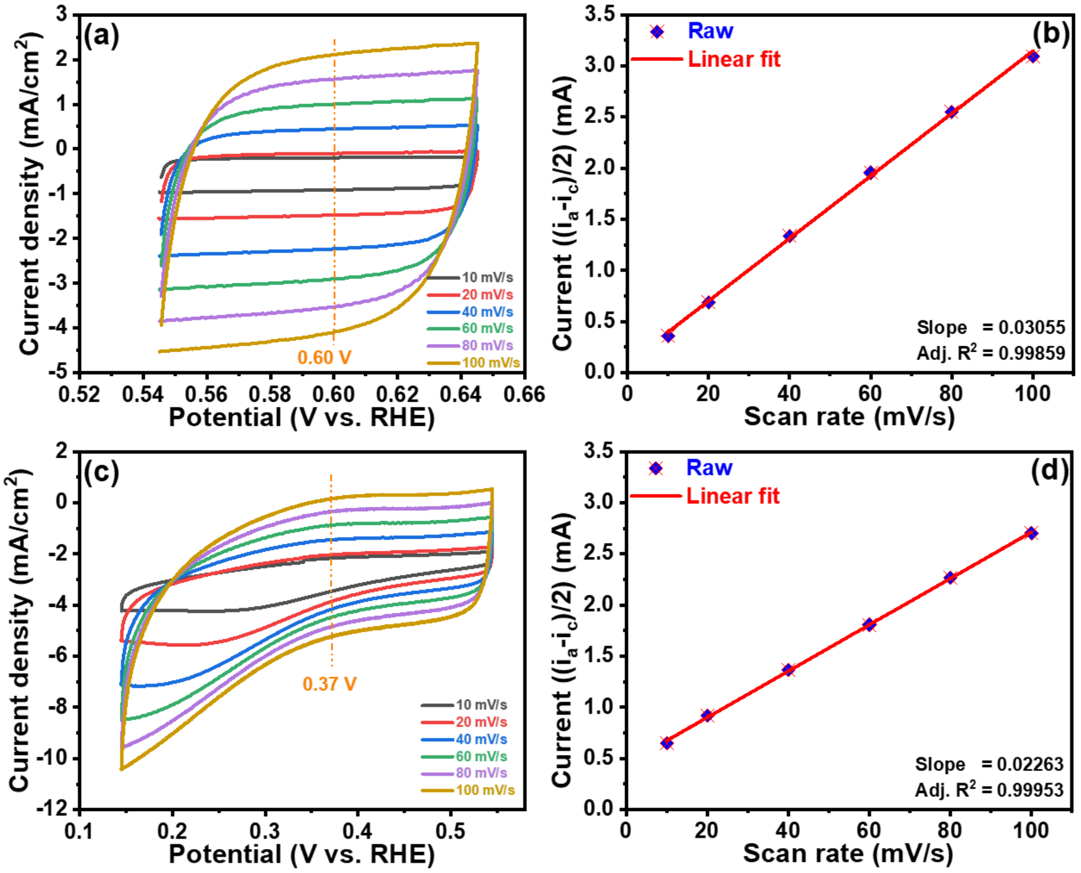

Figure S10. Cyclic voltammograms of (a) L-Pd/rGO, (c) H-Pd/rGO measured at different scan rates in Ar-saturated 1.0 M KOH containing 0.5 M KNO_3; and linear plot of capacitive current of (b) L-Pd/rGO, (d) H-Pd/rGO measured as function of CV scan rate.

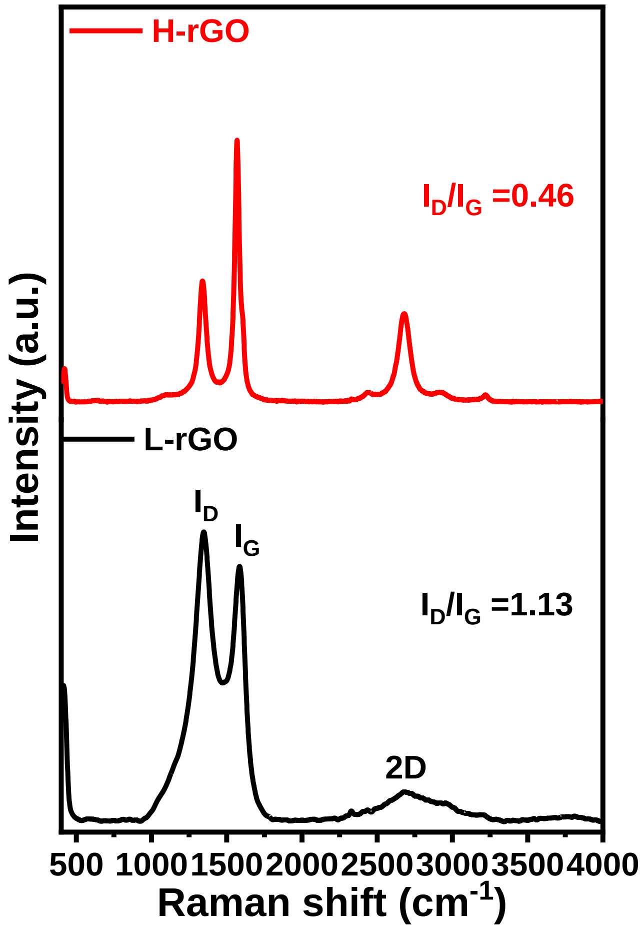

Figure S11. Raman spectra of L-rGO and H-rGO (at 400 □ in air for 2 hrs).

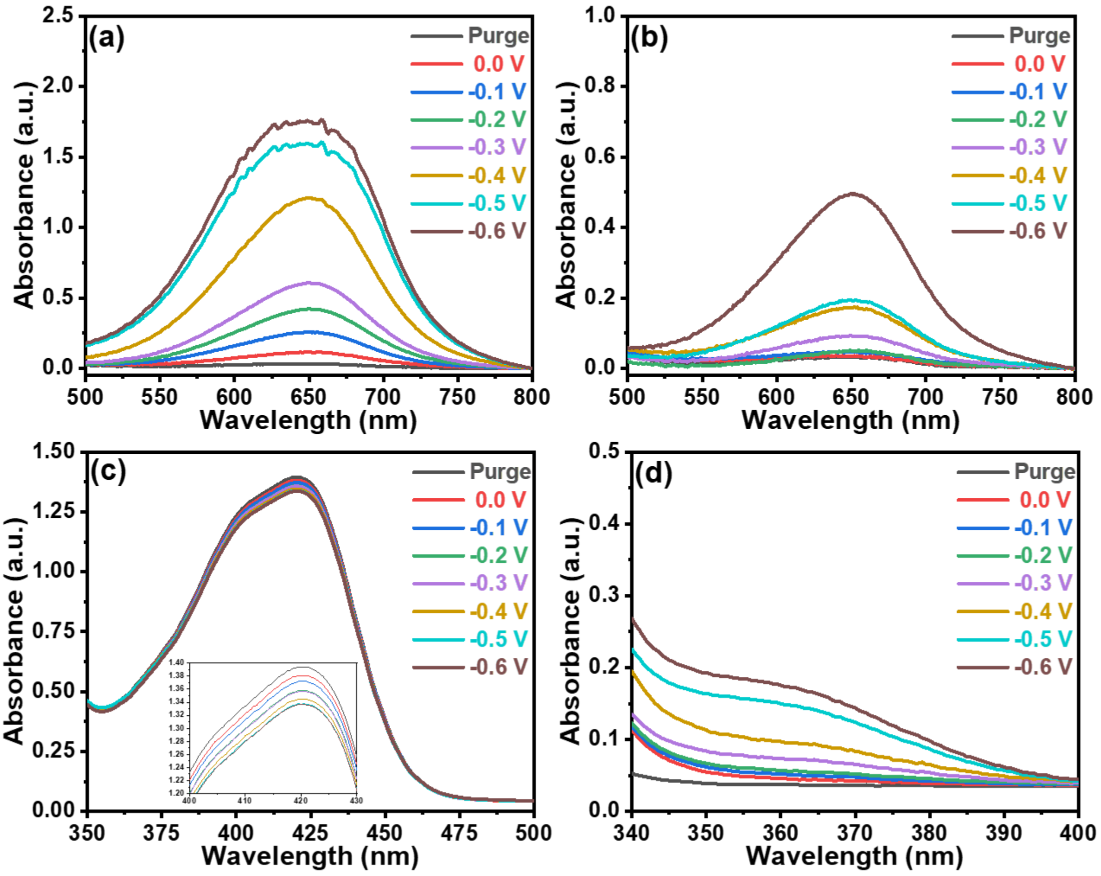

Figure S12. UV-visible spectra of (a) electrolyte (diluted 60 times) in indophenol ammonia quantification, (b) trap (diluted 25 times) in indophenol ammonia quantification, (c) electrolyte in ferricyanide hydroxylamine quantification, and (d) electrolyte in nitrite quantification.

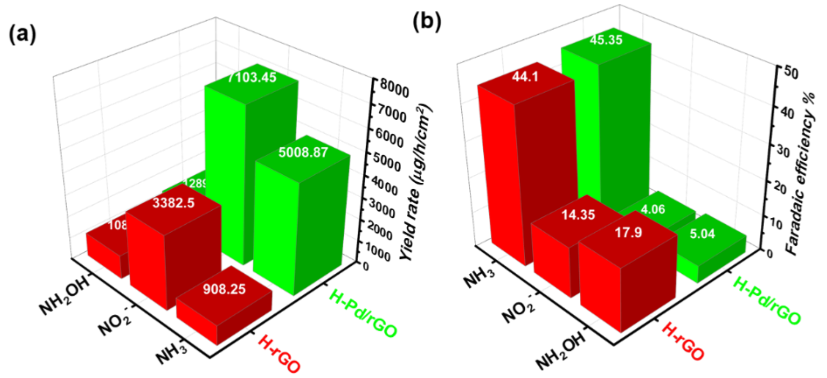

Figure S13. (a) ammonia/nitrite/hydroxylamine yield rate, and (b) faradaic efficiency comparison of H-rGO and H-Pd/rGO composite catalyst at -0.5 V vs. RHE in Ar saturated 1.0 M KOH containing 0.5 M KNO_3 solution.

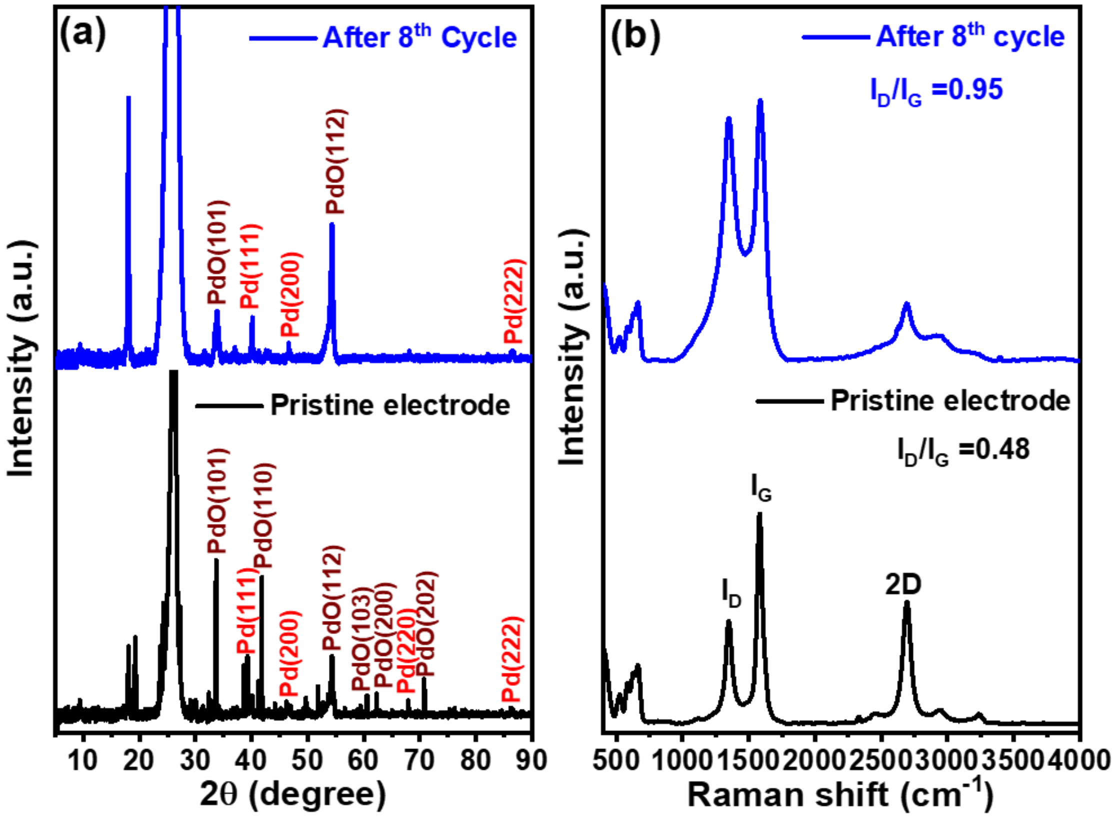

Figure S14. (a) X-ray diffraction pattern, and (b) Raman spectra of L-Pd/rGO coated electrode and after 8 hours of eNO_3RR cycles at -0.4 V vs. RHE.

eNO_3RR cycles	Indophenol blue method (µM)	Ammonia ion selective method (µM)	Deviation (%)
Cycle 1	7706	7884	2.3
Cycle 2	8818	9286	5.3
Cycle 3	8736	9286	6.3
Cycle 4	7731	7447	3.7
Cycle 5	7822	8251	5.5
Cycle 6	8083	7906	2.2
Cycle 7	7678	7089	7.7
Cycle 8	7746	8205	5.9
Cycle 9	7132	7483	4.9
Cycle 10	8261	8530	3.3

Cycle 11	7756	7238	6.7
Cycle 12	6102	6159	0.9
Cycle 13	5538	5846	5.6
Average deviation			4.6

Table S1. Comparison of ammonia concentration analyzed by ammonia ion selective electrode and Indophenol method.

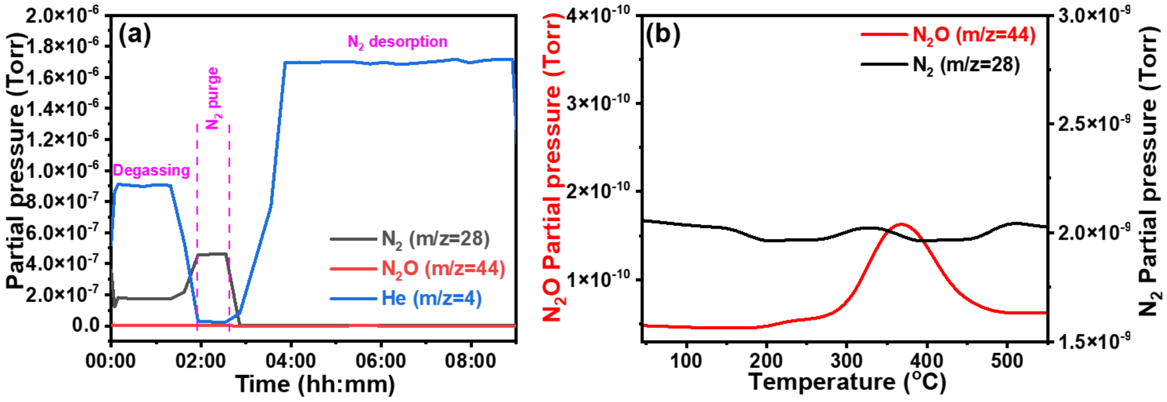

Figure S15. (a) Online mass spectrum recorded during the degassing at 200 °C, nitrogen purging at 50 °C and N_2-TPD experiment of H-Pd/rGO composite, and (b) magnified view of nitrogen and nitrous oxide gas in the region of N_2 desorption.

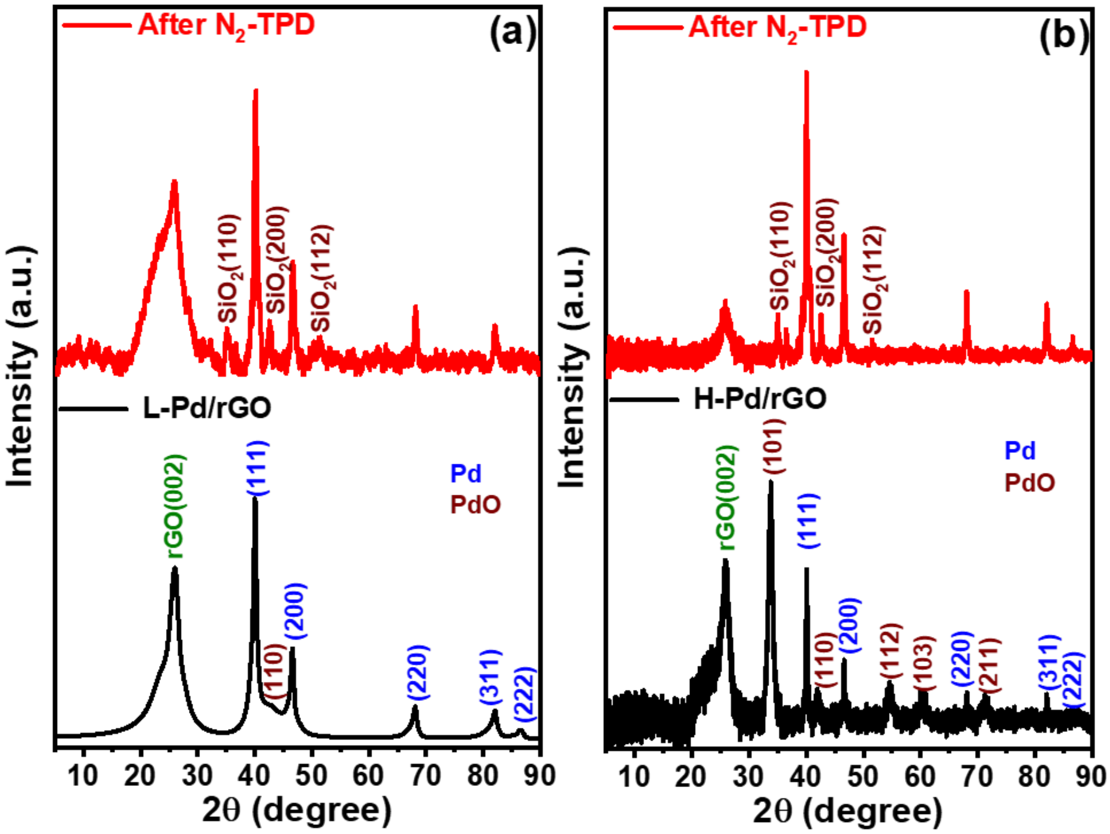

Figure S16. X-ray diffraction pattern of (a) L-Pd/rGO, and (b) H-Pd/rGO composite catalysts after N_2 TPD analysis.

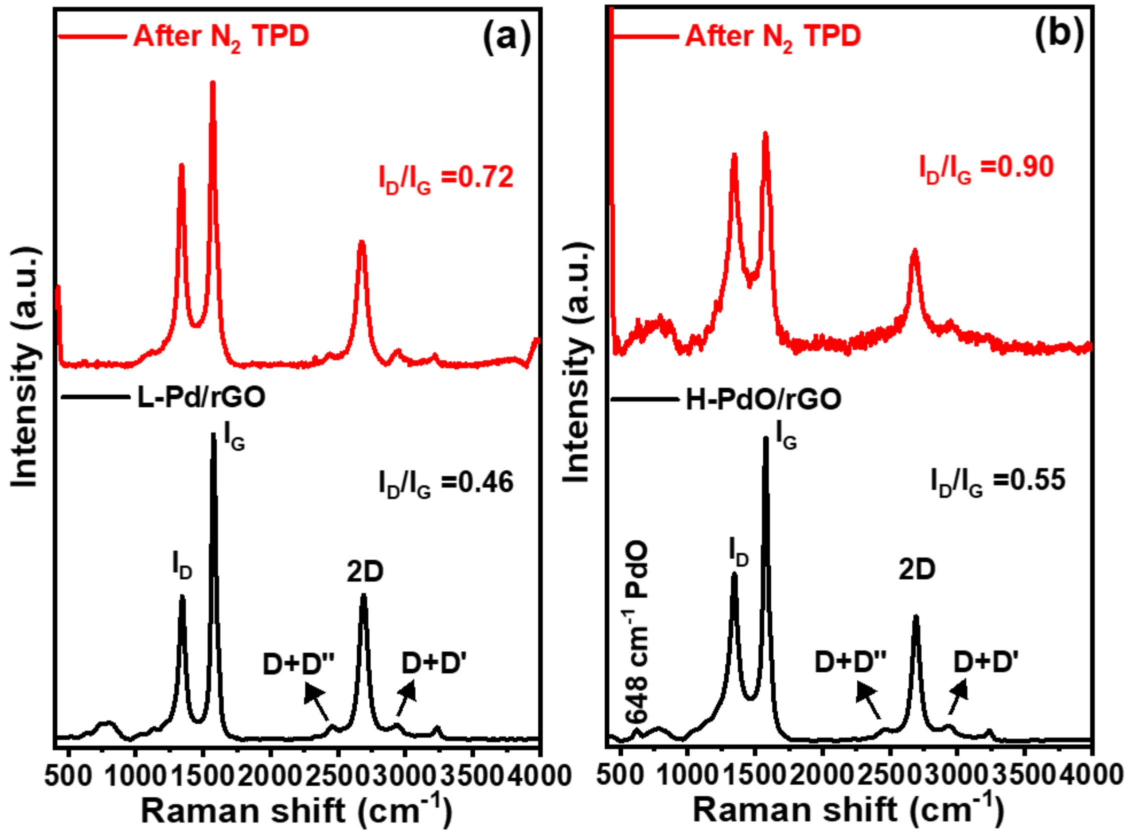

Figure S17. Raman spectra of (a) L-Pd/rGO, and (b) H-Pd/rGO composite catalysts after N_2 TPD analysis.

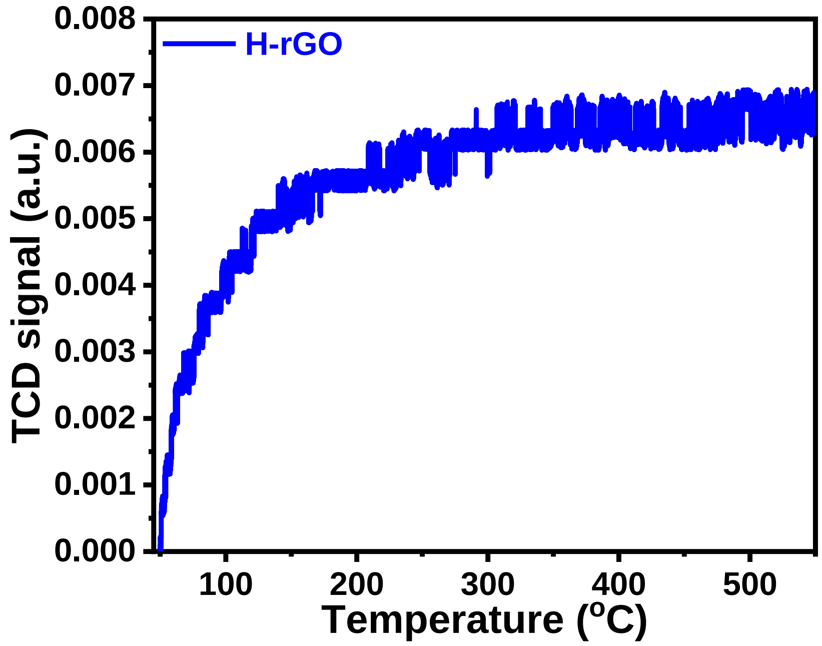

Figure S18. N_2-Temperature programmed desorption profile of H-rGO (at 400 □ in air for 2 hrs).

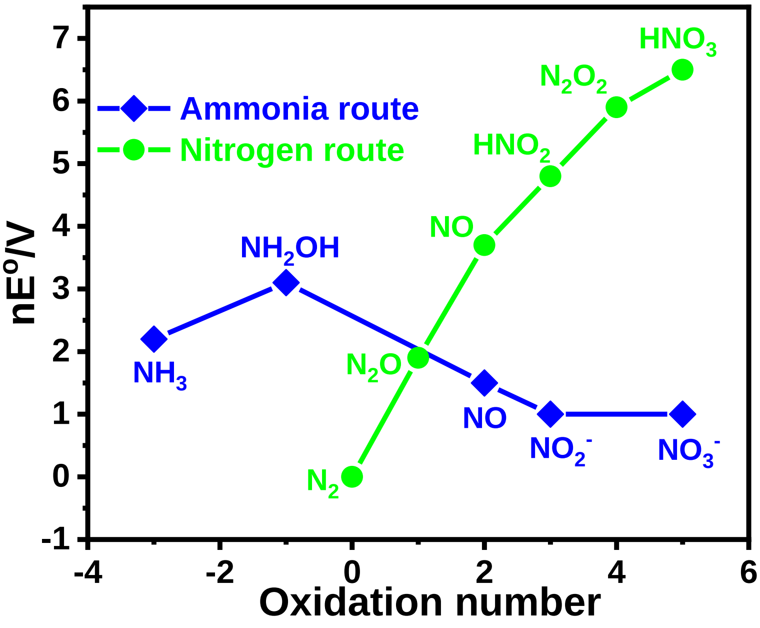

Figure S19. (a) Frost-Ebsworth diagram of nitrogen species at pH 0.0 (green) and (blue) pH 14.0.

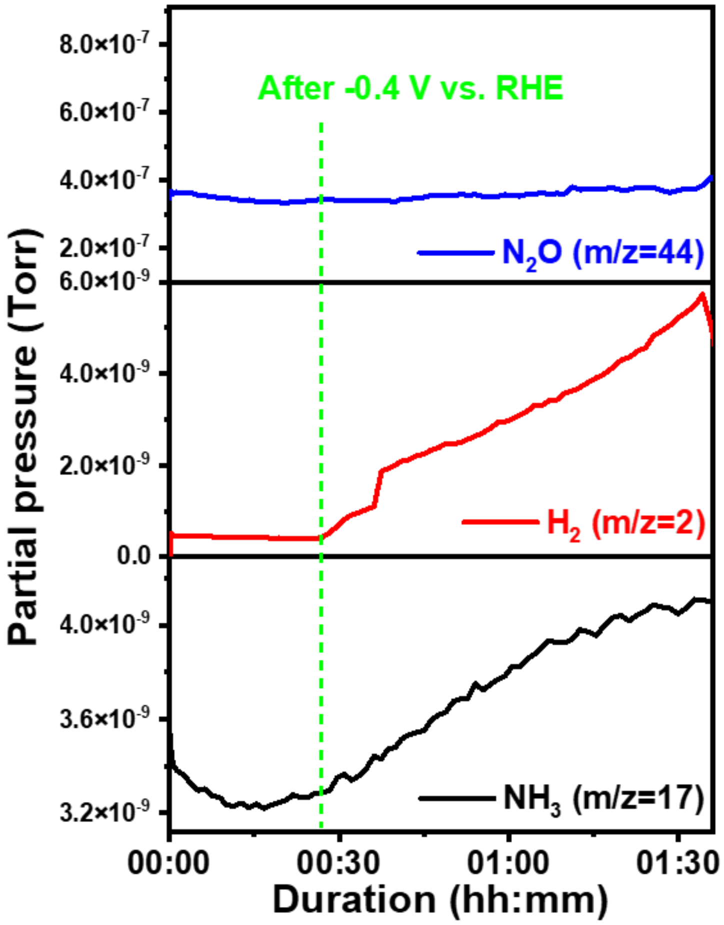

Figure S20. Online mass spectra recorded at -0.4 V vs. RHE for H-Pd/rGO composite catalyst-coated electrode in Ar-saturated 1.0 M KOH solution containing 0.5 M KNO_3.

S. No	Catalyst	Electrolyte, Potential (vs. RHE)	Ammonia yield rate	Faradaic efficiency	Reference
1	Iridium Nanotubes	0.1 M $HClO_4$ + 1 M $NaNO_3$, 0.06 V	921 µg/h/mg_{cat}	84.7%	1
2	RhCu Bimetallic Nanocubes	0.1 M $HClO_4$ + 0.05 M KNO_3, 0.05 V	2.40 mg/h/mg_{cat}	93.7%	2

3	Built-in Electric Field CuCl	0.5 M Na_2SO_4 + 100 mg/L NO_3^-, -1.0 V	1.82 mg/h/cm^2	44.7%	3
4	TiO_2 nanotubes/TiO_{2-x}	0.5 M Na_2SO_4 + 50 mg/L NO_3^-, -0.95 V	0.765 mg/h/cm^2	85.0%	4
5	Co_3O_4-TiO_2/Ti	0.1 M Na_2SO_4 + 50 mg/L NO_3^-, -0.55 V	0.137 mg/h/cm^2	77.1%	5
6	CuO-Co_3O_4/Ti	0.05 M Na_2SO_4 + 100 mg/L NO_3^-, -0.9 V	1.65 mg/h/cm^2	54.5%	6
7	Fe_2O_3/Ti	0.05 M Na_2SO_4 + 100 mg/L NO_3^-, -0.55 V	0.9 mg/h/cm^2	79.1%	7
8	Cu-SAC	50 mM Na_2SO_4 + 50 mg/L NO_3^-, -1.06 V	0.585 mg/h/cm^2	46.7%	8
9	PdCu/Cu_2O hybrids	0.5 M Na_2SO_4 + 100 ppm NO_3^-, -0.80 V	0.191 mmol/h/cm^2	94.3%	9
10	nano-nickel GB	1.0 M NaOH + 1.0 M $NaNO_3$, -0.93 V	15.49 mmol/h/cm^2	93.0%	10
11	10Cu/TiO_{2-x}	0.5 M Na_2SO_4 + 200 ppm NO_3^-, -0.75 V	0.1143 mmol/h/mg	81.3%	11
12	a-RuO_2	0.5 M Na_2SO_4 + 200 ppm NO_3^-, -0.35 V	0.1158 mmol/h/cm^2	97.5%	12
13	Pd-Cu_2O CEO	0.5 M K_2SO_4 + 50 ppm NO_3^-, -1.3 V vs. SCE	925.11 µg/h/mg$_{cat}$	96.6%	13
14	Ru_1Cu_{10}/rGO	1.0 M KOH + 1.0 M KNO_3, -0.05 V	0.38 mmol/h/cm^2	98.0%	14
15	B2CuPd	1.0 M KOH + 1.0 M KNO_3, -0.5 V	6.25 mol/h/g	92.5%	15
16	Cu/CuAu core/shell NCs	1.0 M KOH + 1.0 M KNO_3, -0.5 V	8.47 mol/h/g	85.5%	16
17	V_{Cu}-Au_1Cu SAAs	0.1 M KOH + 7.14 mM NO_3^-, -0.2 V	555 µg/h/cm^2	98.7%	17
18	Pd-NDs/Zr-MOF	0.1 M Na_2SO_4 + 500 ppm NO_3^-, -1.3 V	287.31 mmol/h/g$_{cat}$	58.1%	18
19	Cu@C	1.0 M KOH + 1.0 mM NO_3^-, -0.9 V	469.5 µg/h/cm^2	72.0%	19

20	CoP NRs	0.5 M Na_2SO_4 + 50 mM $NaNO_3$, -0.5 V	30.1 mg/h/mg_{cat}	97.1%	[20]
21	CoP PANSs	0.5 M K_2SO_4 + 0.05 M KNO_3, -0.5 V	19.28 mg/h/mg_{cat}	94.2%	[21]
**22**	**H-Pd/rGO**	**1.0 M KOH + 0.5 M KNO_3, -0.4 V**	**3226.6 ± 35.3 µg/h/cm^2 (14.8 mg/h/mg_{Pd-PdO})**	**52.4 ± 1.6%**	**This work**

Table S2. Comparison of H-Pd/rGO composite catalyst performance with that of previously reported catalysts.

Calculation of standard Gibbs energy

As we detected hydroxylamine in the electrolyte, the possible reaction pathway is proposed below in equations 1 to 5.

$NO_3^-{}_{(aq)} \rightarrow NO_3^-{}_{(ads)}$ (1)

$NO_3^-{}_{(ads)} + H_2O + 2e^- \rightarrow NO_2^-{}_{(ads)} + 2OH^-$ (2)

$NO_2^-{}_{(ads)} + H_2O + e^- \rightarrow NO_{(ads)} + 2OH^-$ (3)

$NO_{(ads)} + 3H_2O + 3e^- \rightarrow NH_2OH_{(ads)} + 3OH^-$ (4)

$NH_2OH_{(ads)} + H_2O + 2e^- \rightarrow NH_3 + 2OH^-$ (5)

The standard Gibbs free energy change of a reaction was calculated using the following equation:

$\Delta G^o_{reaction} = \sum \Delta G^o_f(\text{products}) - \sum \Delta G^o_f(\text{reactants})$

Standard Gibbs Free Energy of Formation (ΔG^o_f)

The standard Gibbs free energies of formation at 25°C (298 K)[22] for the species involved:

- $NO_3^-{}_{(aq)}$: -110.6 kJ/mol

- $NO_2^-{}_{(aq)}$: -9.3 kJ/mol
- $NO_{(g)}$: 86.6 kJ/mol
- $NH_{3(aq)}$: -26.5 kJ/mol
- $NH_2OH_{(aq)}$: 33.5 kJ/mol
- $OH^-{}_{(aq)}$: -157.2 kJ/mol
- $H_2O_{(l)}$: -237.1 kJ/mol

Calculation of ΔG° for Each Reaction

Reaction (1): $NO_3^-{}_{(aq)} \rightarrow NO_3^-{}_{(ads)}$

Assuming $\Delta G^o{}_f(NO_3^-{}_{(ads)}) \approx \Delta G^o{}_f(NO_3^-{}_{(aq)})$:

$\Delta G^O{}_1 = \Delta G_f°{}_{(NO3-(ads))} - \Delta G_f°{}_{(NO3-(aq))} = -110.6 - (-110.6) = 0$ kJ/mol

Reaction (2): $NO_3^-{}_{(ads)} + H_2O + 2e^- \rightarrow NO_2^-{}_{(ads)} + 2OH^-$

$\Delta G_2° = \Delta G_f°{}_{(NO2-(ads))} + 2\Delta G_f°{}_{(OH-)} - \Delta G_f°{}_{(NO3-(ads))} - \Delta G_f°{}_{(H2O)}$

$\Delta G_2° = (-9.3 + (2\times-157.2)) - (-110.6+-237.1)$

$\Delta G_2° = (-9.3-314.4) - (-347.7)$

$\Delta G_2° = -323.4 + 347.7 = 24.3$ kJ/mol

Reaction (3): $NO_2^-{}_{(ads)} + H_2O + e^- \rightarrow NO_{(ads)} + 2OH^-$

$\Delta G_3° = \Delta G_f°{}_{(NO)} + 2\Delta G_f°{}_{(OH-)} - \Delta G_f°{}_{(NO2-)} - \Delta G_f°{}_{(H2O)}$

$\Delta G_3° = (86.6 + (2\times-157.2)) - (-9.3 + (-237.1))$

$\Delta G_3° = (86.6 - 314.4) - (-246.3)$

$\Delta G_3° = -227.8 + 246.3 = 18.6$ kJ/mol

Reaction (4): $NO_{(ads)} + 3H_2O + 3e^- \rightarrow NH_2OH_{(ads)} + 3OH^-$

$\Delta G_4° = \Delta G_{f}°_{(NH2OH)} + 3\Delta G_{f}°_{(OH-)} - \Delta G_{f}°_{(NO)} - 3\Delta G_{f}°_{(H2O)}$

$\Delta G_4° = (33.5 + (3\times-157.2)) - (86.6 + (3\times-237.1))$

$\Delta G_4° = (33.5-471.6)-(86.6-711.3)$

$\Delta G_4° = -438.1-(-624.7) = 186.6$ kJ/mol

Reaction (5): $NH_2OH_{(ads)} + H_2O + 2e^- \rightarrow NH_3 + 2OH^-$

$\Delta G^o_5 = \Delta G_{f}°_{(NH3)} + 2\Delta G_{f}°_{(OH-)} - \Delta G_{f}°_{(NH2OH)} - \Delta G_{f}°_{(H2O)}$

$\Delta G_5° = (-26.5 + (2\times-157.2)) - (33.5-237.1)$

$\Delta G_5° = -340.9 - (-203.6) = -137.3$ kJ/mol

Summary

- Reaction (1): $\Delta G° = 0$ kJ/mol
- Reaction (2): $\Delta G° = 24.3$ kJ/mol
- Reaction (3): $\Delta G° = 18.6$ kJ/mol
- Reaction (4): $\Delta G° = 186.6$ kJ/mol
- Reaction (5): $\Delta G° = -137.3$ kJ/mol

References

(1) Zhu, J. Y.; Xue, Q.; Xue, Y. Y.; Ding, Y.; Li, F. M.; Jin, P.; Chen, P.; Chen, Y. Iridium Nanotubes as Bifunctional Electrocatalysts for Oxygen Evolution and Nitrate Reduction Reactions. *ACS Appl Mater Interfaces* **2020**, *12* (12), 14064–14070. https://doi.org/10.1021/acsami.0c01937.

(2) Ge, Z. X.; Wang, T. J.; Ding, Y.; Yin, S. Bin; Li, F. M.; Chen, P.; Chen, Y. Interfacial Engineering Enhances the Electroactivity of Frame-Like Concave RhCu Bimetallic Nanocubes for Nitrate Reduction. *Adv Energy Mater* **2022**, *12* (15). https://doi.org/10.1002/aenm.202103916.

(3) Sun, W. J.; Ji, H. Q.; Li, L. X.; Zhang, H. Y.; Wang, Z. K.; He, J. H.; Lu, J. M. Built-in Electric Field Triggered Interfacial Accumulation Effect for Efficient Nitrate Removal at Ultra-Low Concentration and Electroreduction to Ammonia. *Angewandte Chemie - International Edition* **2021**, *60* (42), 22933–22939. https://doi.org/10.1002/anie.202109785.

(4) Jia, R.; Wang, Y.; Wang, C.; Ling, Y.; Yu, Y.; Zhang, B. Boosting Selective Nitrate Electroreduction to Ammonium by Constructing Oxygen Vacancies in TiO_2. *ACS Catal* **2020**, *10* (6), 3533–3540. https://doi.org/10.1021/acscatal.9b05260.

(5) Gao, J.; Jiang, B.; Ni, C.; Qi, Y.; Zhang, Y.; Oturan, N.; Oturan, M. A. Non-Precious Co3O4-TiO_2/Ti Cathode Based Electrocatalytic Nitrate Reduction: Preparation, Performance and Mechanism. *Appl Catal B* **2019**, *254*, 391–402. https://doi.org/10.1016/j.apcatb.2019.05.016.

(6) Yang, M.; Wang, J.; Shuang, C.; Li, A. The Improvement on Total Nitrogen Removal in Nitrate Reduction by Using a Prepared CuO–Co_3O_4/Ti Cathode. *Chemosphere* **2020**, *255*. https://doi.org/10.1016/j.chemosphere.2020.126970.

(7) Su, L.; Li, K.; Zhang, H.; Fan, M.; Ying, D.; Sun, T.; Wang, Y.; Jia, J. Electrochemical Nitrate Reduction by Using a Novel Co_3O_4/Ti Cathode. *Water Res* **2017**, *120*, 1–11. https://doi.org/10.1016/j.watres.2017.04.069.

(8) Zhu, T.; Chen, Q.; Liao, P.; Duan, W.; Liang, S.; Yan, Z.; Feng, C. Single-Atom Cu Catalysts for Enhanced Electrocatalytic Nitrate Reduction with Significant Alleviation of Nitrite Production. *Small* **2020**, *16* (49). https://doi.org/10.1002/smll.202004526.

(9) Yin, H.; Chen, Z.; Xiong, S.; Chen, J.; Wang, C.; Wang, R.; Kuwahara, Y.; Luo, J.; Yamashita, H.; Peng, Y.; Li, J. Alloying Effect-Induced Electron Polarization Drives Nitrate Electroreduction to Ammonia. *Chem Catalysis* **2021**, *1* (5), 1088–1103. https://doi.org/10.1016/j.checat.2021.08.014.

(10) Zhou, J.; Wen, M.; Huang, R.; Wu, Q.; Luo, Y.; Tian, Y.; Wei, G.; Fu, Y. Regulating Active Hydrogen Adsorbed on Grain Boundary Defects of Nano-Nickel for Boosting Ammonia

Electrosynthesis from Nitrate. *Energy Environ Sci* **2023**, *16* (6), 2611–2620. https://doi.org/10.1039/d2ee04095f.

(11) Zhang, X.; Wang, C.; Guo, Y.; Zhang, B.; Wang, Y.; Yu, Y. Cu Clusters/TiO_{2-x} with Abundant Oxygen Vacancies for Enhanced Electrocatalytic Nitrate Reduction to Ammonia. *J Mater Chem A Mater* **2022**, *10* (12), 6448–6453. https://doi.org/10.1039/d2ta00661h.

(12) Wang, Y.; Li, H.; Zhou, W.; Zhang, X.; Zhang, B.; Yu, Y. Structurally Disordered RuO_2 Nanosheets with Rich Oxygen Vacancies for Enhanced Nitrate Electroreduction to Ammonia. *Angewandte Chemie - International Edition* **2022**, *61* (19). https://doi.org/10.1002/anie.202202604.

(13) Xu, Y.; Ren, K.; Ren, T.; Wang, M.; Wang, Z.; Li, X.; Wang, L.; Wang, H. Ultralow-Content Pd in-Situ Incorporation Mediated Hierarchical Defects in Corner-Etched Cu_2O Octahedra for Enhanced Electrocatalytic Nitrate Reduction to Ammonia. *Appl Catal B* **2022**, *306*. https://doi.org/10.1016/j.apcatb.2022.121094.

(14) Gao, W.; Xie, K.; Xie, J.; Wang, X.; Zhang, H.; Chen, S.; Wang, H.; Li, Z.; Li, C. Alloying of Cu with Ru Enabling the Relay Catalysis for Reduction of Nitrate to Ammonia. *Advanced Materials* **2023**. https://doi.org/10.1002/adma.202202952.

(15) Gao, Q.; Pillai, H. S.; Huang, Y.; Liu, S.; Mu, Q.; Han, X.; Yan, Z.; Zhou, H.; He, Q.; Xin, H.; Zhu, H. Breaking Adsorption-Energy Scaling Limitations of Electrocatalytic Nitrate Reduction on Intermetallic CuPd Nanocubes by Machine-Learned Insights. *Nat Commun* **2022**, *13* (1). https://doi.org/10.1038/s41467-022-29926-w.

(16) Gao, Q.; Yao, B.; Pillai, H. S.; Zang, W.; Han, X.; Liu, Y.; Yu, S. W.; Yan, Z.; Min, B.; Zhang, S.; Zhou, H.; Ma, L.; Xin, H.; He, Q.; Zhu, H. Synthesis of Core/Shell Nanocrystals with Ordered Intermetallic Single-Atom Alloy Layers for Nitrate Electroreduction to Ammonia. *Nature Synthesis* **2023**, *2* (7), 624–634. https://doi.org/10.1038/s44160-023-00258-x.

(17) Zhang, Y.; Chen, X.; Wang, W.; Yin, L.; Crittenden, J. C. Electrocatalytic Nitrate Reduction to Ammonia on Defective Au_1Cu (111) Single-Atom Alloys. *Appl Catal B* **2022**, *310*. https://doi.org/10.1016/j.apcatb.2022.121346.

(18) Jiang, M.; Su, J.; Song, X.; Zhang, P.; Zhu, M.; Qin, L.; Tie, Z.; Zuo, J. L.; Jin, Z. Interfacial Reduction Nucleation of Noble Metal Nanodots on Redox-Active Metal-Organic Frameworks for High-Efficiency Electrocatalytic Conversion of Nitrate to Ammonia. *Nano Lett* **2022**, *22* (6), 2529–2537. https://doi.org/10.1021/acs.nanolett.2c00446.

(19) Song, Z.; Liu, Y.; Zhong, Y.; Guo, Q.; Zeng, J.; Geng, Z. Efficient Electroreduction of Nitrate into Ammonia at Ultralow Concentrations Via an Enrichment Effect. *Advanced Materials* **2022**, *34* (36). https://doi.org/10.1002/adma.202204306.

(20) Hong, Q. L.; Zhou, J.; Zhai, Q. G.; Jiang, Y. C.; Hu, M. C.; Xiao, X.; Li, S. N.; Chen, Y. Cobalt Phosphide Nanorings towards Efficient Electrocatalytic Nitrate Reduction to Ammonia. *Chemical Communications* **2021**, *57* (88), 11621–11624. https://doi.org/10.1039/d1cc04952f.

(21) Jia, Y.; Ji, Y. G.; Xue, Q.; Li, F. M.; Zhao, G. T.; Jin, P. J.; Li, S. N.; Chen, Y. Efficient Nitrate-to-Ammonia Electroreduction at Cobalt Phosphide Nanoshuttles. *ACS Appl Mater Interfaces* **2021**, *13* (38), 45521–45527. https://doi.org/10.1021/acsami.1c12512.

(22) Lide, D. R.; Baysinger, G.; Berger, L. I.; Goldberg, R. N.; Kehiaian, H. V; Kuchitsu, K.; Roth, D. L.; Zwillinger, D. *CRC Handbook of Chemistry and Physics, Internet version **2005**,* http://www.hbcpnetbase.com.
